# Accelerating the discovery of insensitive high-energy-density materials by a materials genome approach

**DOI:** 10.1038/s41467-018-04897-z

**Published:** 2018-06-22

**Authors:** Yi Wang, Yuji Liu, Siwei Song, Zhijian Yang, Xiujuan Qi, Kangcai Wang, Yu Liu, Qinghua Zhang, Yong Tian

**Affiliations:** 1 Institute of Chemical Materials, China Academy of Engineering Physics (CAEP), Mianyang, 621000 China; 2 Sichuan Co-Innovation Center for New Energetic Materials, Southwest University of Science and Technology, Mianyang, 621010 China

## Abstract

Finding new high-energy-density materials with desired properties has been intensely-pursued in recent decades. However, the contradictory relationship between high energy and low mechanical sensitivity makes the innovation of insensitive high-energy-density materials an enormous challenge. Here, we show how a materials genome approach can be used to accelerate the discovery of new insensitive high-energy explosives by identification of “genetic” features, rapid molecular design, and screening, as well as experimental synthesis of a target molecule, 2,4,6-triamino-5-nitropyrimidine-1,3-dioxide. This as-synthesized energetic compound exhibits a graphite-like layered crystal structure with a high measured density of 1.95 g cm^−3^, high thermal decomposition temperature of 284 °C, high detonation velocity of 9169 m s^−1^, and extremely low mechanical sensitivities (impact sensitivity, >60 J and friction sensitivity, >360 N). Besides the considered system of six-member aromatic and hetero-aromatic rings, this materials genome approach can also be applicable to the development of new high-performing energetic materials.

## Abstract (web summary)

The synthesis of explosive materials that are stable, highly dense, and have low sensitivity to external stimuli is a challenge. Here, the authors use a genomic approach to accelerate the discovery of insensitive high explosive molecules with good detonation and low sensitivity properties.

## Introduction

Since the invention of black powder in China and the era of nitroglycerin brought by Alfred Nobel, high explosives have contributed enormously to the progress and prosperity of mankind^1,2^. With the rapid development of modern high-energy-density materials (HEDMs), a large variety of high-performing energetic materials have been developed and continue to play an overwhelming role in some of the today’s fantastic engineering projects and space exploration^3–19^. However, the increasing severity of safety issues in both military and civil applications renders the menu of available insensitive high explosive (IHE) molecules vanishingly small^20,21^. To date, the only approved IHE molecule that meets the criteria of United States Department of Energy is 1,3,5-triamino-2,4,6-trinitrobenzene (TATB)^22^, a high-density aromatic compound first synthesized in 1888^23^. Owing to the extremely low sensitivities to external stimuli, TATB is also described as “wood explosive”^24,25^ and usually preferred for applications where extreme safety is required, such as the explosive formulations in nuclear weapons and low burning rate propellant components for space exploration. However, the detonation energy of TATB is relatively low, around 65% of the widely used high explosive 1,3,5,7-tetranitro-1,3,5,7-tetrazocane (HMX)^26^. Over the past decades, a few low-sensitivity HEDMs, such as 1,1-diamino-2,2-dinitroethene(FOX-7) and 2,6-diamino-3,5-dinitropyrazine-1-oxide (LLM-105), have been reported as potential TATB replacements, but their safety issues are still far away from industrial standard of IHE molecules^27–29^. Ideally, new IHE molecules are expected to have the TATB’s sensitivity and the HMX’s energy. But in most cases, the high detonation performances of HEDMs are accompanied with the undesirable high sensitivity to external stimuli such as impact, shock, spark, friction, and heat. This contradictory relationship between high energy and low sensitivity makes the development of new high-performing IHE molecules a long-standing challenge in chemical sciences^30^.

As compared to the revolutionary advances in other functional materials, the progressive process of new insensitive HEDMs is painfully slow, probably due to that the traditional synthesis of high explosives still relies primarily on scientific intuition and trial-and-error^31,32^. In 2011, the US government launched the Materials Genome Initiative (MGI), which aims to reduce the development time by providing the infrastructure and training that innovators need to discover new advanced materials in a more efficient and economical way. In recent years, the materials genome approach has been applied in new material innovations and helped scientists successfully discover new alloy materials^33,34^, metal-organic frameworks (MOF)^35,36^, and inorganic porous materials^37,38^. These fascinating advances suggest the success of materials genome approach for the new materials discovery.

Here, we show how the materials genome approach has been used for the rapid design and structural screening of new IHE molecules with desired properties, as this allows us to quickly choose potential IHE molecule candidates for the experimental synthesis. In this approach, we first identify three key “genetic” features of IHE molecules from large amounts of component and structural information. The second step involves the rapid design and screening of candidate molecules with the guidance of identified “genetic” features. Third, we experimentally synthesized the screened target molecule. The property studies showed that the as-synthesized energetic compound (also named as ICM-102) was a promising IHE molecule with a high measured density (1.95 g cm^−3^), high thermal decomposition temperature (284 °C), high detonation velocity (9169 m s^−1^), and extremely low sensitivities (like TATB) to external stimuli such as impact, friction, and electrostatic spark.

## Results

### Identification of key “genetic” features of IHE molecules

**Fig. 1:**
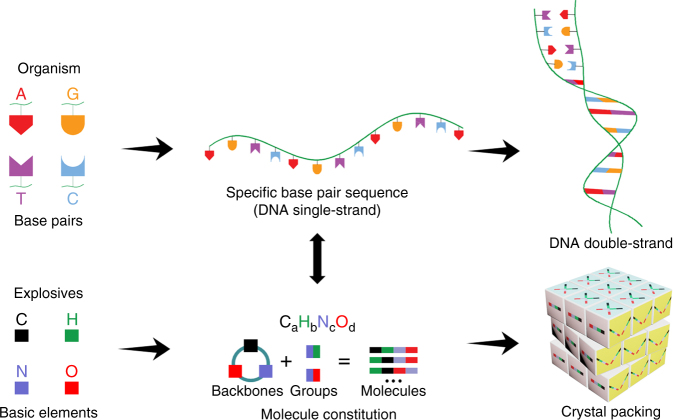
Illustration of possible materials genomes of organic explosives. Comparison of materials genome approach for organic explosives with that of an organism

Before the computationally-guided design and rapid screening of new IHE molecules by a materials genome approach, the first challenge in this work is the identification of key material “genetic” features from those reported IHE molecules. Since high density and low sensitivity are the two most important properties for high-performing IHE molecules, we attempt to explore the “genetic code” of typical HEDMs, i.e., to reveal the intrinsic relationships between materials’ structure (e.g., molecular constitution and crystal packing) and properties (e.g., high density and low sensitivities) from large bodies of experimental data. It is well known that four basic chemical bases (A, T, G, C) provide the super-complex genetic information of organism by some specific arranged sequences and intermolecular interactions (Fig. 1). An extraordinary coincidence is that most HEDMs are generally composed of four basic elements (C, H, N, O) and their permutation and combination decide the original molecular constitution. For a given molecular constitution, various HEDMs molecules can be designed by rational combination of molecular backbones and functional groups. Accordingly, various crystal packing structures of one designed molecule are formed by the self-assembly of extensive intermolecular interactions (e.g., hydrogen bonds and π–π stacking) (Fig. 1). Therefore, the basic element constitution, molecular structure, and crystal packing manners together determine the energy-density and mechanical sensitivities of HEDMs. Based on above analysis, we are interested in identifying the key structural features (also called “genetic” information) of desired IHE molecules from both molecular and crystal aspects, respectively.

**Fig. 2:**
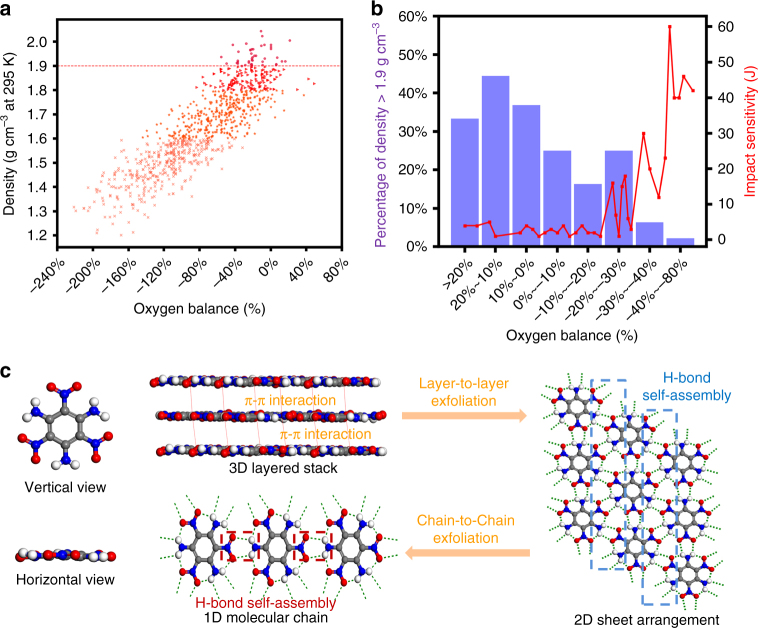
Identification of key “genetic” features of IHE molecules. **a** Relationship between crystal densities and CO_2_-based oxygen balance of the organic compounds constituted by C, H, N, O atoms. **b** Percentage of the density above 1.90 g cm^−3^ in different CO_2_-based oxygen balance ranges (blue histogram) and impact insensitivities of some representative explosives with density above 1.90 g cm^−3^ in different CO_2_-based oxygen balance ranges (red dots). **c** Planar molecular configuration and step-to-step exfoliation of TATB crystal packing from the π–π interaction-driven three-dimensional layered stacking to a two-dimensional sheet arrangement and a hydrogen-bond self-assembled one-dimensional molecular chain

From the view of molecular constitution, we first investigated the crystal densities of more than 1000 neutral organic compounds based on C, H, N, and O elements from Cambridge Structural Database (CSD) and found an important law that the crystal densities of CHNO-based organic molecules are linearly related to their CO_2_-based oxygen balances. In general, high CO_2_-based oxygen balances usually indicate high crystal density (Fig. 2a). For example, the results of statistical analysis demonstrated that a quite high percentage (>30%) of CHNO-based compounds with a CO_2_-based oxygen balance values above zero exhibited the densities of >1.90 g cm^−3^ at room temperature (Fig. 2b, column chart). Undesirably, these compounds with both high density and high oxygen balances are usually sensitive to external stimuli with impact sensitivities of <10 J (Fig. 2b, red dots), indicating the contradictory relationship between high energy and low sensitivity. These results are also consistent with the experimental observations of previous studies^39^. By contrast, only 3% of CHNO-based compounds have densities above 1.90 g cm^−3^ for the ones with the CO_2_-based oxygen balances between −80 and −40%, meanwhile they are all less sensitive to external stimuli with impact sensitivities of >30 J (Fig. 2b, red dots). Through the above analyses, we conclude that a relatively low CO_2_-based oxygen balance value in the range of −80 to −40% is desirable for the HEDMs’ insensitivity, which can be identified as the first key “genetic” information of new IHE molecules. It is worth noting that the only approved IHE molecule (TATB) by Department of Energy has a CO_2_-based oxygen balance of −55.8%, which falls right in this “genetic” category.

**Table 1:** Physical properties of ICM-102 and comparison with TATB, RDX, HMX, LLM-105, and FOX-7

	ICM-102	TATB	RDX	HMX	LLM-105	FOX-7
*T*_d_^a^ (°C)	284	360	210	279	342	220
*ρ*^b^ (g cm^−3^)	1.95	1.94	1.80	1.90	1.91	1.88
Δ_*f*_*H*_m_ (kJ mol^−1^)	−8.1^c^	−139.5^d^	86.3^d^	116.1^d^	−12.0^d^	−118.9^d^
*P* (GPa)	34.3^e^	32.4^l^	34.9^e^	39.2^e^	33.4^m^	35.9^e^
*ν*_D_ (m s^−1^)	9169^f^	8114^l^	8878^f^	9221^f^	8560^m^	9000^f^
IS^g^ (J)	>60	>60	7.5	7.5	28.7	24.7
FS^h^ (N)	>360	>360	120	120	>360	>360
*H*_50_^i^ (cm)	320	320	26	32	117	126
*E*_50_^j^ (J)	1.85	2.27	0.151	0.099	1.02	1.92
OB^k^ (%)	−55.45	−55.81	−21.61	−21.69	−37.04	−21.61

^a^Decomposition temperature (exothermic peak)

^b^Measured density by a gas pycnometer at 298 K

^c^Calculated heat of formation

^d^Measured heats of formation

^e^Detonation pressure calculated using EXPLO5/6.02

^f^Detonation velocity calculated using EXPLO5/6.02

^g^Impact sensitivity evaluated by a standard BAM fall-hammer

^h^Friction sensitivity evaluated by a BAM friction tester

^i^Tested by 2.5 kg drop hammer

^j^Electrostatic spark sensitivity

^k^Oxygen balance based on CO_2_ for C_a_H_b_N_c_O_d_:OB (%) = 1600×(*d*−*a*−*b*/2)/*M*_*w*_

^l^Ref. ^9^

^m^Ref. ^61^

With the aim of further understanding the relationships between molecular structure and insensitivity, we selected eight representative HEDMs with the features of insensitivity or low sensitivity and studied their structural features that affect sensitivity at the molecular level (Supplementary Fig. 1). After a systematic analysis of their molecular structures, several common features of low-sensitivity HEDMs can be addressed: (a) a large degree of internal charge delocalization in the molecule and the facilitation of π–π stacking in the crystal can significantly provide a desensitizing effect for a given HEDM molecule, so it is helpful to choose the aromatic or hetero-aromatic ring as the parent framework for new IHE molecules; (b) the energetic groups (e.g., nitro group and N^+^–O^**−**^ group) and stabilizing groups (e.g., amino group) must be reasonably introduced into the parent molecular framework (e.g., alternating amino and nitro arrangement), ensuring the resulting HEDM molecules with a planar conformation and high symmetry. Based on the above analyses, we think that the planar aromatic (or hetero-aromatic) structures with high symmetry (e.g., C_1v_ or better symmetries) can also be identified as the second key “genetic” feature of IHE molecules.

In addition to molecular constitution and structures, understanding how crystal packing manners affect the HEDMs’ sensitivity is also of vital importance. Due to unique high crystal density (1.94 g cm^−3^) and extremely low sensitivity to external stimuli, the example of TATB is still given for illustration (Fig. 2c). It is known that TATB has a graphitic-like crystal structure with low free space in the lattice, in which the TATB layers form a close-packing by intermolecular π–π stacking. In light of crystal packing, the graphitic-like layered crystal structure of TATB plays a vital role in its extremely low sensitivity to external stimuli (such as impact, shock, spark, friction, and heat)^40^. In this regard, it is recognized that the HEDMs’ graphitic-like crystal structure facilitates to translate less heat from shear and slip than other crystal packing structures (such as wavelike stacking, crossing stacking, and mixing stacking), with the concomitant reduced formation in hot spots. The similar results are observed in all other graphite-like HEDMs (e.g., DAAF and DAAzF) (Supplementary Fig. 2a), which have both high energy (detonation velocity > 8600 m/s) and very low impact sensitivities (IS > 60 J)^41^.

People have realized the importance of graphite-like crystal structure for the insensitivity of IHE molecules. However, the knowledge on how to design an explosive molecule with graphite-like layered crystal structure is limited, and only a few graphite-like energetic compounds have been reported so far (Supplementary Fig. 2a). Take TATB as an example, there are several factors playing the indispensable roles in the formation of graphite-like crystal structure (Fig. 2c), which include: (a) a planar molecular configuration; (b) extensive hydrogen bonding interactions, which provide additional driving force to form one-dimensional molecular chain; (c) reasonable self-assembly of one-dimensional chains to two-dimensional molecular sheets by intermolecular hydrogen bonds; and (d) two-dimensional molecular sheets fabricate the three-dimensional layered packing by intermolecular π–π stacking. Only after all the above conditions are satisfied, the designed molecules have the possibility to form the graphite-like layered crystal packing. A typical example is 5-amino-3-nitro-1,2,4-triazole (ANTA). Although ANTA has a planar molecular configuration and can further assemble into one-dimensional molecular chain by intermolecular hydrogen bonds, it finally fails to form a graphite-like layered packing due to the absence of suitable hydrogen bonds for the formation of two-dimensional molecular sheet (Supplementary Fig. 3). Therefore, we conclude that the graphite-like crystal packing can be identified as the third “genetic” feature of IHE molecules.

Through systematically analyzing basic element constitution, molecular structures, and the manners of crystal packing, three key “genetic” features of potential IHE molecules have been extracted. These “genetic” features provide an important physical basis (i.e., the boundary conditions) for the following rapid design and screening of new IHE molecules.

### Rapid design and screening of new IHE molecules

**Fig. 3:**
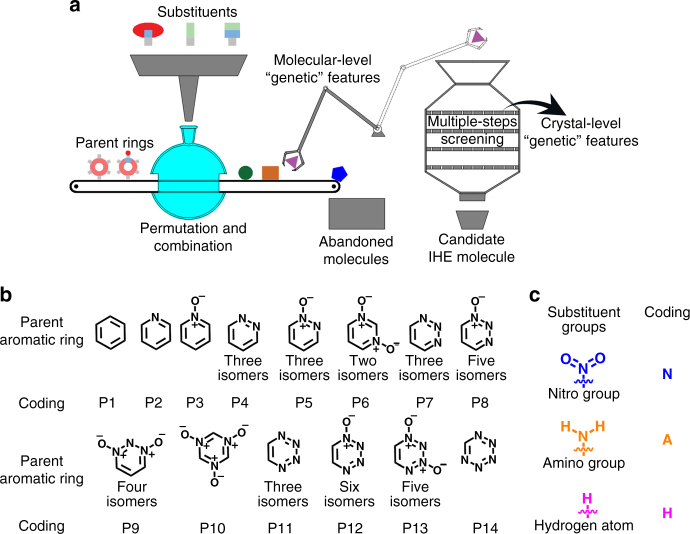
Rapid design of new IHE molecules. **a** Illustration of computer-assisted rapid screening process of new IHE molecules. **b** Selected parent six-member aromatic rings and their gene coding. **c** Selected substituent groups and their gene coding

**Fig. 4:**
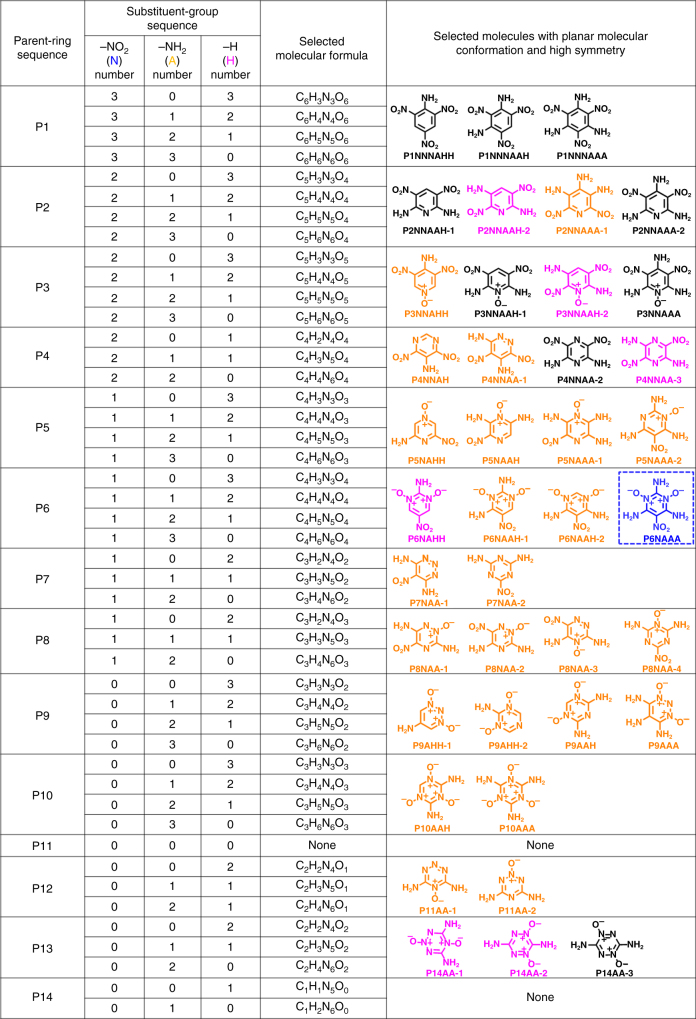
Rapid screening of new graphite-like IHE molecules. Selected molecular formula and molecules by the identified key “genetic” features of IHE molecules (black molecules have been widely studied and some of them are indeed graphite-like crystal structures; pink molecules probably have graphite-like crystal structures; yellow molecules cannot have graphite-like crystal structures; blue molecules probably have graphite-like crystal structures with synthetic feasibility)

Aiming at the rapid design and screening of new target IHE molecules, we first tried to construct a library of several favorable structural segments (i.e., aromatic parent rings and substituent groups) and then enumerated all possible molecular formulas and structures by computationally-guided combinations between aromatic parent rings and substituent groups, as illustrated in Fig. 3a. Subsequently, by virtue of three key “genetic” features of IHE molecules, we assign a set of boundary conditions to each molecule in the library, which allow us to quickly screen the candidate structures that may meet our requirements (Fig. 3a). In our approach, 14 kinds of six-member aromatic rings (including benzene rings and *N*-hetero-aromatic rings) are selected and coded as P1 to P14, the selected substituent groups include nitro group (–NO_2_), amino group (–NH_2_), and hydrogen atom (–H) (Fig. 3b). By means of self-compiled Java scripts, we enumerated all the combinatorial possibility between 14 selected six-member aromatic rings and three substituent groups, and 176 suited molecular formulas were screened out (see Supplementary Note 1). When we applied the first key “genetic” feature (low CO_2_-based oxygen balance of −80 to −40%) as the boundary to these candidate molecules, the suitable molecular formulas decreased from 176 to 45 (see Supplementary Note 2 and Fig. 4), and 402 molecules in the library passed this screen with the consideration of isomers (Supplementary Figs. 4–10). To further narrow the range of candidate IHE molecules, we continue to apply the second key “genetic” features as the boundary condition to the screened molecules. Among 402 candidates, only 40 molecules show the planar conformation with high symmetry under the screening constraint of the second key “genetic” features (Fig. 4).

**Fig. 5:**
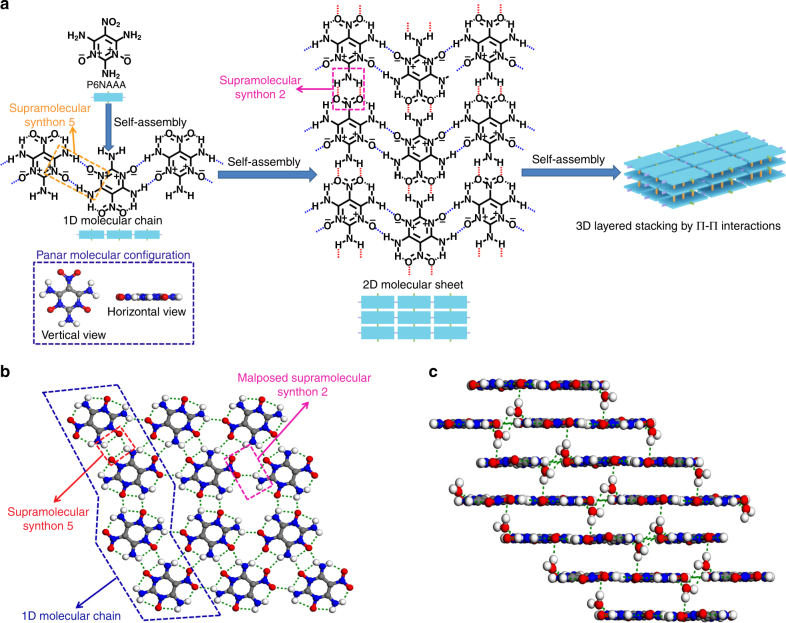
Crystal structure of ICM-102. **a** The probable three-dimensional graphite-like topological structure of P6NAAA by self-assembly of supramolecular synthon 5 and supramolecular synthon 2. **b** Two-dimensional molecular sheet of ICM-102 in crystal. **c** Three-dimensional graphite-like layered packing of ICM-102 containing water molecules

To further screen the potential candidates, we also introduced the supramolecular synthons as a useful tool to guide the prediction of intermolecular interactions and screening of new graphite-like IHE molecules. It is known that supramolecular synthons represent some characteristic supramolecular spatial arrangements of intermolecular interactions^42^ and widely used for the crystal structure prediction in crystal engineering^43–45^. Here 15 main supramolecular synthons have been identified from the reported graphite-like high explosives (Supplementary Fig. 2b). Among the selected 40 molecules with planar conformation and high symmetry, only seven molecules (pink and blue molecules in Fig. 4) have the potential to form the graphite-like crystal structures with the aids of supramolecular synthons (Fig. 5a and Supplementary Figs. 11–13), except some reported graphite-like explosives, such as P1NNNAAA (also known as TATB), P2NNAAA-2 (also known as TIBMUM in Supplementary Fig. 2a)^46^, P3NNAAH-1 (also known as TIBMIA in Supplementary Fig. 2a)^46^, and P3NNAAA (also known as TIBMOG in Supplementary Fig. 2a)^46^ (the black molecules, Fig. 4). We further calculated the theoretical crystal density and found that P6NAAA (the blue molecule in Fig. 4) shows the highest theoretical crystal density (1.91 g cm^−3^) among seven molecules (Supplementary Note 4 and Supplementary Fig. 21). In this molecule, the alternate arrangement of energetic groups (nitro group and N^+^–O^**−**^ group) and stabilizing groups (amino group) on the pyrimidine ring makes it a planar conformation with high symmetry (Fig. 5a). In addition, it has a high probability of constructing the two-dimensional layered structure by means of supramolecular synthon 2 and supramolecular synthon 5. As a result, this candidate molecule has great potential to form the graphite-like crystal packing through π–π interactions between two-dimensional layers (Fig. 5a). The theoretical crystal predictions also demonstrate that this compound has the potential to form a graphite-like layered crystal structure through supramolecular synthon 4 and supramolecular synthon 5 (Supplementary Note 5 and Supplementary Fig. 22). Therefore, P6NAAA is finally screened as the target IHE molecule that is worthy of our synthetic effort.

When looking at this screening process as a whole, a clear goal-directed thinking can be found: a library of basic structural segments (i.e., parent rings and substituents) is constructed with the guidance of designing new HEDMs molecules; all the chemical structures with specified molecular formulas are computationally enumerated; rapid screening is carried out using three identified key “genetic” features of IHE molecules as the boundary conditions; and finally, the candidate molecule with desired structural features is filtered out.

### Synthesis and crystal structure

To validate our predictions that the selected P6NAAA would be a high-density compound with graphite-like crystal structure, we turned to its experimental synthesis (note: here we named the P6NAAA as ICM-102). When we searched the structure of ICM-102 by SciFinder Web, we found that its synthesis was first described in 1968 by Delia et al.^47^, then followed by Hollins et al. and Millar et al. in 1996 and 2004, respectively^48,49^. However, we found that the structural characterization information of ICM-102 is quite limited in three references, and crystal structural information is also absent^47–49^. In addition, the previous literatures reported that the density of ICM-102 was just 1.81 g cm^−3^^48,49^, which is obviously lower than our predicted one (1.91 g cm^−3^) (Supplementary Note 4 and Supplementary Fig. 21). The curiosity prompted us to continue the synthesis and property studies on the ICM-102. To our surprise, when repeating the reported methods of using H_2_O_2_/CF_3_COOH to oxidize the precursor of 5-nitro-2,4,6-triaminopyrimidine (or 5-nitroso-2,4,6-triaminopyrimidine), we failed to obtain the target molecule of ICM-102. After carefully analyzing their synthetic routes, we found that the post-treatment operations reported previously are unreasonable due to the absence of an acid–base neutralization step (Supplementary Note 6 and Supplementary Fig. 23). Subsequently, our crystal structural analysis also confirmed that the pale-yellow precipitate from highly acidic H_2_O_2_/CF_3_COOH oxidation system is actually trifluoroacetate salt of ICM-102 (one ICM-102 molecule is protonated with two CF_3_COOH molecules) (Supplementary Table 1 and Supplementary Fig. 14), which needs to be neutralized to afford the neutral target molecule. In contrast, the reported post-treatment operations of recrystallization from water^47^ or washing with propan-2-ol^49^ cannot produce the neutral ICM-102, and their obtained products are a mixture of ICM-102, its trifluoroacetate salt, and some unknown impurities (Supplementary Note 6).

After slightly adjusting the ratio between raw material and oxidizer and adding the acid–base neutralization step in post-treatment (see Methods part), we successfully obtained the neutral ICM-102 samples with yellow color (Supplementary Fig. 15a). The elemental analysis of the as-prepared yellow samples is N% of 38.01 and C% of 21.65, which are almost identical to the theoretical values of ICM-102 monohydrate (N% 38.18 and C% 21.82 of ICM-102·H_2_O). Single-crystal X-ray diffraction also reveals the formation of ICM-102 monohydrate (Supplementary Table 2). ICM-102 crystallizes in the P21/c space group and each ICM-102 molecule interacted with five nearby molecules through hydrogen bonds to form a planar layer structure (Fig. 5b). The layer structures were further packing along [001] direction to construct graphite-like supramolecular structure (Fig. 5c) by π–π interactions. The average interlamellar spacing (3.194 Å) of ICM-102 is larger than those of other layered explosives (e.g., TATB, FOX-7, LLM-105, and NTO) (Supplementary Fig. 16), which is probably due to the existing water molecules between layers. In the intra-layer self-assembly of ICM-102, the predicted supramolecular synthon 5 (namely the hydrogen bonds between N^+^–O^−^ and H–N) assembles to form one-dimensional molecular chain with hydrogen distances around 2.0 Å (Fig. 5b and Supplementary Fig. 16b). The one-dimensional molecular chain further assembles into two-dimensional molecular sheet with a malposed supramolecular synthon 2, which comes from the interference of water molecules (Fig. 5b). The hydrogen distances between two chains are around 2.2 Å (Supplementary Fig. 16b). The successful validation of graphite-like layered structure of ICM-102 implies that supramolecular synthons are indeed a useful tool to predict crystal structures of new HEDMs.

Since the water molecules are inserted into two supramolecular layers of ICM-102 (Fig. 5c), a higher density of ICM-102 can be achieved when the inter-layer water molecules are removed. First, we tried to remove the crystal water by a simple heating method because the dehydration temperature of ICM-102·H_2_O is 178 °C (Supplementary Fig. 17). When the as-prepared sample of ICM-102 was heated to 150 °C under vacuum (5 torr) for 5 h, the color of ICM-102 turned to orange from bright yellow with a measured density of 1.92 g cm^−3^. Elemental analysis showed that the sample is close to that of anhydrous ICM-102 (Supplementary Figs. 15a, b), indicating that the heating treatment can remove most crystal waters from the ICM-102 monohydrate. After multiple tries, we finally found an efficient method to prepare the anhydrous ICM-102, i.e., the recrystallization of ICM-102 monohydrate from hot DMSO/EtOAc (see Methods part). The color of anhydrous ICM-102 became much deeper than the heated sample at 150 °C, and its measured density is high to 1.95 g cm^−3^ (Supplementary Fig. 15c), demonstrating our success of obtaining anhydrous ICM-102 samples.

### Thermal properties

**Fig. 6:**
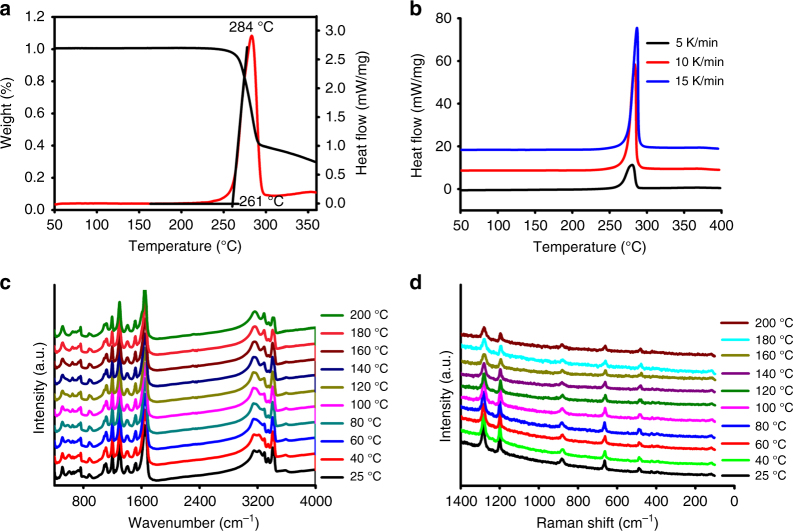
Thermostability of ICM-102. **a** Differential scanning calorimeter (DSC) and thermal gravimetric analyzer (TG) curves of ICM-102. **b** Differential scanning calorimeter (DSC) curves of ICM-102 with different heating rates. **c** In-situ variable-temperature infrared spectra of ICM-102. **d** In-situ variable-temperature Raman spectra of ICM-102

The thermal decomposition temperature (*T*_d_) of ICM-102 was determined by differential scanning calorimeter (DSC) and thermal gravimetric analyzer (TG) measurements with exothermic peak at 284 °C (Fig. 6a), which is much superior to RDX and FOX-7 (210 °C and 220 °C, respectively) and close to HMX (279 °C) (Table 1). In order to well understand the thermal decomposition process of ICM-102, we have further investigated its non-isothermal kinetic apparent activation energy (*E*_a_) of the exothermic decomposition reaction by a multiple heating method (Kissinger method^50^ and Ozawa method^51^).

After multiple DSC measurements with different heating rates (Fig. 6b), the calculated *E*_a_ by Kissinger and Ozawa methods are almost identical, with the values of 448.7 kJ mol^−1^ and 435.5 kJ mol^−1^, respectively (Supplementary Table 3). The high apparent activation energy (*E*_a_) further supports the high thermal stability of ICM-102 (*T*_d_: 284 °C). In addition, we also calculated the self-accelerating decomposition temperature (*T*_SADT_) and critical temperature of thermal explosion (*T*_b_) of ICM-102^52,53^, which are another two significant parameters to ensure safe storage and process operations for energetic materials. The results show that both *T*_SADT_ and *T*_b_ are very high with the values of 252.5 °C and 257.7 °C, respectively (Supplementary Table 4), indicating that thermal stability of ICM-102 was high enough for safe storage and formulation operations. Besides, in-situ characterization techniques of variable-temperature infrared and Raman spectra have been applied to evaluate the thermal stability of ICM-102. When ICM-102 was heated from room temperature to 200 °C, their infrared and Raman spectra remained almost unchanged (Fig. 6c, d), indicating that ICM-102 have no observable thermal decomposition until 200 °C.

### Solubility

As we noted above, ICM-102 exhibits a relatively poor solubility in water. With regard to its practical application, there is a need to find an appropriate solvent for its crystal engineering as it is necessary to obtain high-quality explosive crystals. Hence, we measured the solubility of ICM-102 in water and common organic solvents. Eight common organic solvents and water were tested, and the solubility results are listed in Supplementary Table 5. As compared to the solubility of 220 mg of ICM-102 in 100 ml water at 25 °C, ICM-102 exhibited a much lower solubility of <10 mg per 100 ml in most organic solvents including ethanol, methanol, acetone, acetonitrile, ethyl acetate, and dichloromethane. Even in DMSO, ICM-102 was only slightly soluble at 25 °C (20 mg per 100 ml) and partially soluble (400 mg per 100 ml) at 130 °C (see Methods part). We speculate that extensive hydrogen bonding interactions play a crucial role in low solubility of ICM-102 in most organic solvents at room temperature.

### Detonation properties

After evaluating the physicochemical properties of ICM-102, including density, solubility, and thermal properties, our attention has been turned to its detonation properties. The heat of formation (Δ_*f*_*H*) of ICM-102 was first calculated by the isodesmic reaction approach using Gaussian 09 (Revision D.01) suite program^54^, and was estimated to be −8.1 kJ mol^−1^ (see Supplementary Note 3). Using the measured ambient temperature density (1.95 g cm^−3^) and the calculated heat of formation, the detonation velocity (*ν*_D_) and the detonation pressure (*P*) of ICM-102 were evaluated using EXPLO5 (v6.02) program^55^. As shown in Table 1, the calculated detonation velocity and detonation pressure of ICM-102 are 9169 m s^−1^ and 34.3 GPa, respectively, which are much superior to those of TATB (8114 m s^−1^ and 32.4 GPa) (Table 1). Except HMX (*ν*_D_ = 9221 m s^−1^), the detonation velocity of ICM-102 is also superior to those of RDX (*ν*_D_ = 8878 m s^−1^), LLM-105 (*ν*_D_ = 8560 m s^−1^), and FOX-7 (*ν*_D_ = 9000 m s^−1^). Due to the relatively low OB (−55.45%), the detonation pressure of ICM-102 (34.2 GPa) is lower than those of HMX (39.2 GPa), FOX-7 (35.9 GPa), and RDX (34.9 GPa). From the view of energy level, ICM-102 has excellent detonation properties (*ν*_D_=9169 m s^−1^ and *P* = 34.3 GPa) and is comparable to HMX.

### Sensitivities

The goal of our research is to obtain the desired IHE molecule with HMX’s energy and TATB’s sensitivity. Having evaluated the energetic properties of ICM-102, our attention was particularly shifted to investigate its sensitivities towards external stimuli. To ensure the accuracy and comparability of impact sensitivities, we employed two kinds of professional methods (i.e., standard BAM and characteristic height (*H*_50_) methods, respectively) for every test. The results showed that the impact sensitivity of ICM-102 is extremely low (IS > 60 J and *H*_50_ = 320 cm), which are comparable to TATB and much lower than typical low-sensitivity explosives (LLM-105 and FOX-7) (Table 1). The friction sensitivity of ICM-102 also falls in the insensitivity range with the value of >360 N (Table 1). In addition, the electrostatic spark sensitivity of ICM-102 is also quite low (*E*_a_ = 1.85 J), just slightly lower than TATB (*E*_a_ = 2.27 J) (Table 1). Since ICM-102 has extremely low sensitivities to external stimuli, we attempted to use a standard plate-dent test to evaluate its detonation performance. As expected, we found that ICM-102 can be successfully initiated by a standard plate-dent test with an obvious dent in the steel anvil (Supplementary Fig. 18), demonstrating its promise as an IHE molecule.

**Fig. 7:**
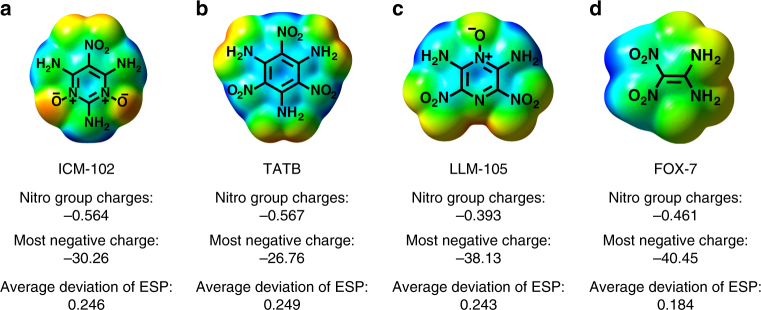
Molecular simulations of ICM-102 and typical IHE molecules. **a** Molecular electrostatic potential (ESP) and nitro group charge of ICM-102. **b** Molecular electrostatic potential (ESP) and nitro group charge of TATB. **c** Molecular electrostatic potential (ESP) and nitro group charge of LLM-105. **d** Molecular electrostatic potential (ESP) and nitro group charge of FOX-7

The extremely low sensitivity of ICM-102 is further explained from both molecular and crystal levels. At the molecular level, it is known that lower nitro group charges^56,57^, more negative ESP^58^, and an average deviation of ESP approaching 0.25^59^ result in low sensitivity of high explosives. As compared with TATB, ICM-102 has a more negative ESP, a comparable nitro group charge and average deviation of ESP caused by alternate positive and negative potentials (Fig. 7a, b). Though the negative ESP of LLM-105 and FOX-7 are lower than that of ICM-102, their two factors of nitro group charges and average deviation of ESP are not comparable to ICM-102 (Fig. 7a, b, d). Therefore, the extremely low sensitivity of ICM-102 can be explained by its unique electronic structure, including low group charge (−0.564), very negative ESP (−30.26), and excellent average deviation of ESP (0.246).

From the view of crystal structure, we noted that the intermolecular hydrogen bond distances of ICM-102 in its two-dimensional layers are very short with an average value of 2.143 Å, even shorter than other layered explosives (such as TATB, LLM-105, FOX-7, and NTO) (Supplementary Fig. 16). This implies that the intermolecular hydrogen bond interactions of ICM-102 are extremely strong and it has a strong tendency to self-assemble into two-dimensional layered structure. As a result, the layer-to-layer buffer and slip of ICM-102 could well consume external energy inputs to make the formation of local hot spots difficult^60^. Therefore, both the unique electronic structure distribution and graphite-like layered structure of ICM-102 are responsible for its extremely low sensitivities to external stimuli.

## Discussion

This study describes how a materials genomic approach is used to accelerate the discovery of a promising IHE molecule with HMX’s energy and TATB’s sensitivity. By rationalizing the relationships between structure and property from large amounts of experimental data, we first identified and extracted key materials “genetic” features that determine the high density and insensitivity of IHE molecules. Second, we performed a computationally-guided molecular design and rapid screening, which includes the library construction of molecular segments and the structural selection of candidate molecules that are worthy of synthetic efforts using the identified “genetic” features as the filtration conditions. Third, we experimentally validated our prediction through a facile and low-cost synthesis of ICM-102 from commercially available raw materials in high yields. Single crystal XRD analysis reveals that ICM-102 has the expected graphite-like crystal structure. The results of property studies demonstrate that ICM-102 has a high measured density of 1.95 g cm^−3^, high thermal decomposition temperature of 284 °C, high detonation velocity of 9169 m s^−1^, and extremely low sensitivities to external stimuli, showing good agreement between prediction and experiment. The success of this materials genome approach not only discovers a promising IHE molecule of ICM-102, but also opens a new avenue to develop new high-performing HEDMs by expanding this approach to multiple or fused hetero-aromatic ring systems.

## Methods

### General

Although ICM-102 shows the surprisingly low sensitivities to external mechanical stimuli (such as impact and friction), the fuming nitric acid, concentrated sulfuric acid, and trifluoroacetic acid are used in the synthesis process. Caution, safety equipments such as protective gloves and coats, face shield, and explosion-proof baffle are recommended.

### Materials

2,4,6-Triamine-pyrimidine (98%), fuming nitric acid (98%), concentrated sulfuric acid (98%), trifluoroacetic acid, and hydrogen peroxide aqueous (30%) were all purchased from commercial sources and used without further purification.

### Product characterization

^1^H and ^13^C NMR spectra were measured at 600 MHz (Bruker AVANCE 600) with DMSO-d_6_ as the solvent. High-resolution mass spectra were performed on a Shimadzu LCMS-IT-TOF mass spectrometer using electrospray ionization (ESI). Elemental analysis was performed on a Vario Micro cube elemental analyzer. Thermal property measurements were carried out on a TGA/DSC Mettler Toledo calorimeter equipped with an auto cool accessory. Impact and friction sensitivity measurements were made using a standard BAM Fall hammer and a BAM friction tester. X-ray powder diffraction (PXRD) analysis was performed on a Bruker D8 Advance X-ray powder diffractometer. FTIR spectra at different temperatures were recorded (transmission mode) using a NICOLET 6700 FTIR (Thermo, Waltham, MA, USA), with a deuterated triglycine sulfate (DTGS) detector. Raman measurements were performed with an FT-Raman spectrometer (DXR smart Raman). The heats of formation and detonation properties were calculated with the Gaussian 09 and Explo5 (version 6.02) software, respectively.

### Synthesis of ICM-102

The target compound ICM-102 was obtained by two-step reactions (nitrification and oxidation) from 2,4,6-triamine-pyrimidine. The nitrification of 2,4,6-triamine-pyrimidine: 4 ml fuming nitric acid (98%) was slowly added into 4 ml concentrated sulfuric acid (98%) in ice-water bath, then 1 g (8.0 mmol) 2,4,6-triamine-pyrimidine was added by portions into the mixed acid. Maintained in ice-water bath for 20 min, the reaction mixture poured into ice-water and filtered to obtain a pale-yellow precipitate. After washing with a large amount of water and acetone, the pale-yellow solid was dried under vacuum, and pure 2,4,6-triamino-5-nitropyrimidine was obtained as a pale-yellow powder (1.29 g, 95% yield). ^1^H NMR (600 MHz, DMSO-d_6_): *δ* ppm: 9.13 (s, 2H, NH), 8.84 (s, 2H, NH), 7.92 (s, 2H, NH); ^13^C NMR (151MHz, DMSO-d_6_): *δ* ppm: 156.78, 153.49, 107.36 (the images of ^1^H and ^13^C NMR spectra are provided in Supplementary Fig. 19). The oxidation of 2,4,6-triamino-5-nitropyrimidine: 1 g (5.9 mmol) 2,4,6-triamino-5-nitropyrimidine was dissolved in 10 ml trifluoroacetic acid at room temperature, then 2.5 ml hydrogen peroxide aqueous (30%) was slowly added. Maintained at room temperature for 8 h, pale-yellow precipitate was filtered and dissolved in 20 ml water. When the solution was neutralized to pH around 7 with NaHCO_3_, a lot of yellow precipitate formed and filtered out. After washing with small amount of water, the yellow solid was dried naturally as ICM-102 monohydrate (0.59 g, 60% yield).

The preparation of anhydrous ICM-102 sample: 0.4 g yellow ICM-102 monohydrate was first dissolved in 100 ml DMSO at 130 °C, and 100 ml ethyl acetate was added into above solution rapidly at this temperature. After that, the mixed solution was cooled to room temperature. The orange sample was filtered and dried under vacuum with an isolated yield of 62.5% (0.25 g). *T*_dec_: 284 °C. ^1^H NMR (600 MHz, DMSO-d_6_): *δ* ppm: 9.01 (s, 4H, NH), 8.45 (s, 2H, NH); ^13^C NMR (151 MHz, DMSO-d_6_): *δ* ppm: 149.52, 146.81, 106.19; ESI-HRMS: *m*/*z* calcd for [M+H]^+^: 203.0529, found: 203.0530 (the images of ^1^H NMR, ^13^C NMR, and ESI-HRMS spectra are provided in Supplementary Fig. 20); elemental analysis calcd (%) for C_4_H_6_N_6_O_4_ (202.0451): C 23.77, H 2.99, N 41.58; found: C 23.63, H 2.91, N 41.44.

### Data availability

The data that support the findings of this study are available from the corresponding authors on request. X-ray coordinates from the crystal structure determinations have been deposited with the Cambridge Crystallographic Data Centre (CCDC), under deposition numbers 1816963 and 1831055. These data can be obtained free of charge from The Cambridge Crystallographic Data Centre via www.ccdc.cam.ac.uk/data_request/cif.

## Electronic supplementary material

Supplementary Information
