# Supplementary material for: Accelerating the discovery of insensitive high-energy-density materials by a materials genome approach
Source: Nat Commun. 2018 Jun 22;9:2444. doi: 10.1038/s41467-018-04897-z (PMC6015015; doi:10.1038/s41467-018-04897-z)
Supplement: Supplementary file 1 — Supplementary Information [file 41467_2018_4897_MOESM1_ESM.pdf]

Supplementary Information

Accelerating the discovery of insensitive high-energy-density materials by a materials genome approach

Wang *et. al.*

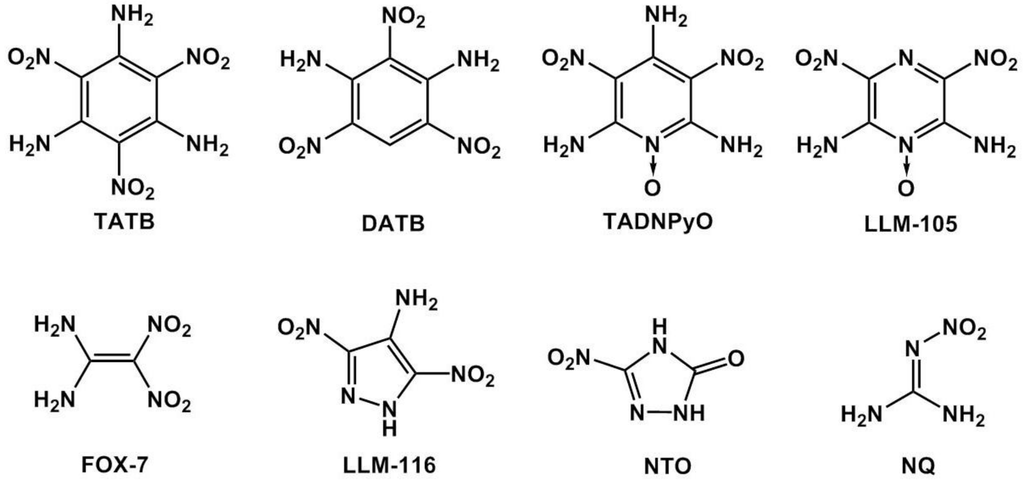

Explosives \ Features	π-conjugated parent structure	Alternating amino and nitro arrangements	High molecular symmetry
TATB	+	+	+
DATB	+	+	+
TADNPyO	+	+	+
LLM-105	+	+	+
FOX-7	+	+	+
LLM-116	+	+	+
NTO	+	-	+
NQ	+	+	+

Supplementary Figure 1. Identification of common molecular features of insensitive and low-sensitive high explosives. Chemical structures and common molecular features of some insensitive and low-sensitive high explosives.

By analyzing these representative insensitive and low-sensitive high explosives, we find that they share some common molecular features including π-conjugated parent structure, alternating amino and nitro arrangements and high molecular symmetry (e.g., C_{1v} or better symmetries).

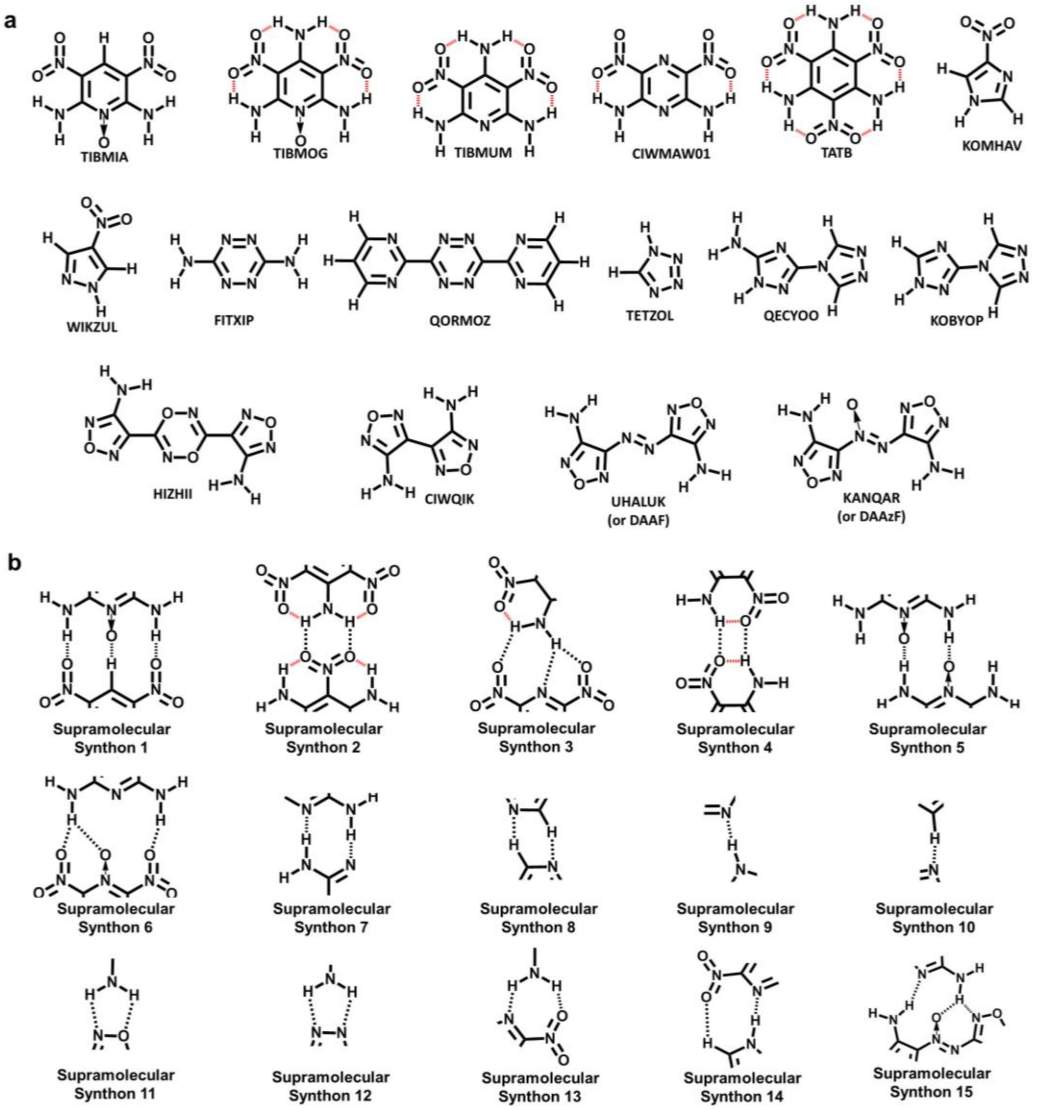

Supplementary Figure 2. Identification of supramolecular synthons of graphite-like explosives. **a** Chemical structures of reported graphite-like explosives from Cambridge Structural Database (CSD). **b** Main supramolecular synthons of these graphite-like explosives (red dot represents intramolecular hydrogen bonds, black dot represents intermolecular hydrogen bonds).

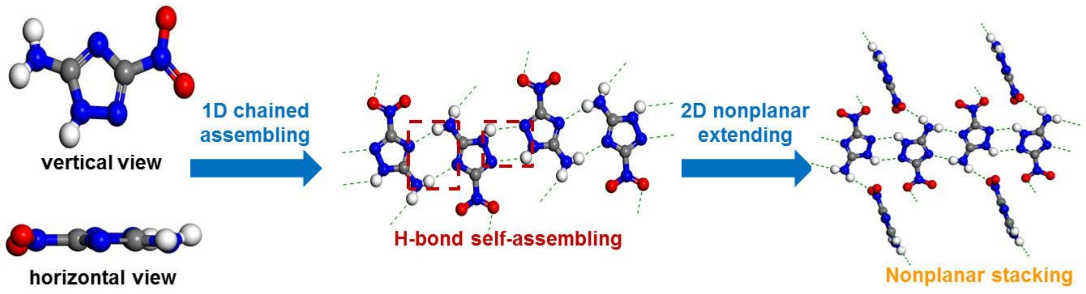

Supplementary Figure 3. Self-assembly of 5-amino-3-nitro-1, 2, 4-triazole in crystal. Molecular configuration and self-assembly in crystal of 5-amino-3-nitro-1, 2, 4-triazole (ANTA).

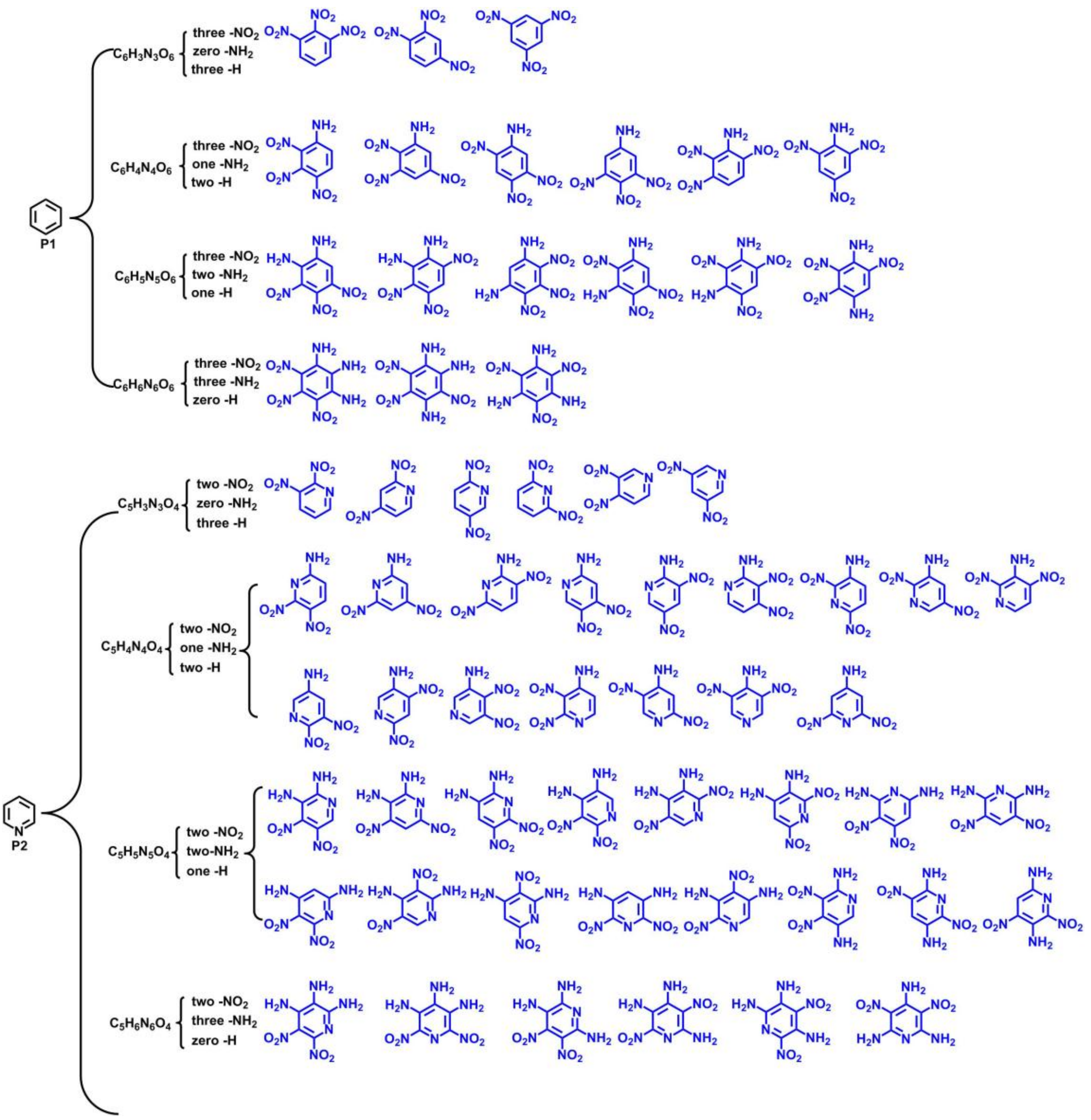

Supplementary Figure 4. Screened molecules by a combination between P1-P2 parent rings and three substituent groups ($-NH_2$, $-NO_2$ and -H) with Oxygen Balance constrain.

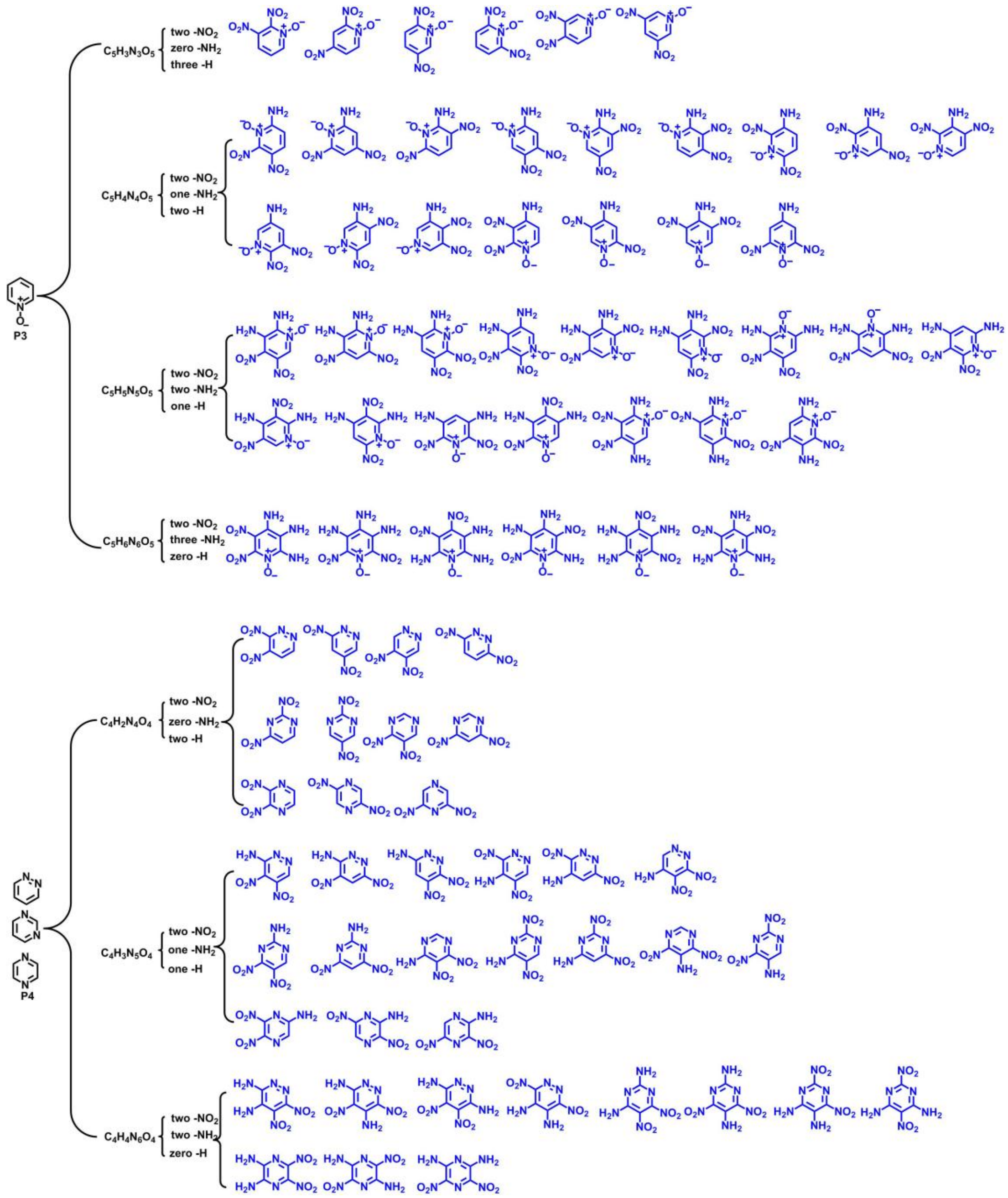

Supplementary Figure 5. Screened molecules by a combination between P3-P4 parent rings and three substituent groups (-NH_2, -NO_2 and -H) with Oxygen Balance constrain.

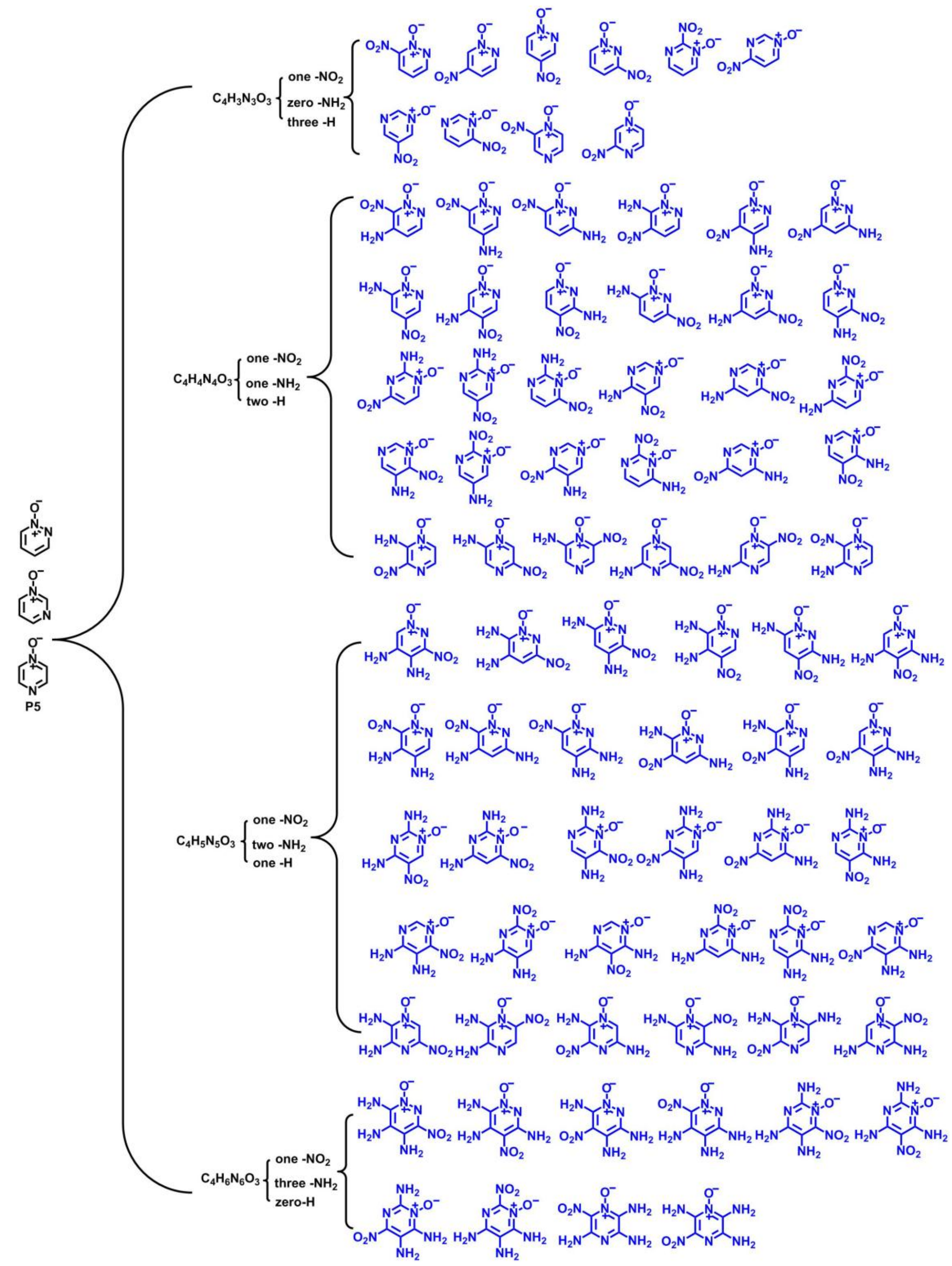

Supplementary Figure 6. Screened molecules by a combination between P5 parent ring and three substituent groups (-NH_2, -NO_2 and -H) with Oxygen Balance constrain.

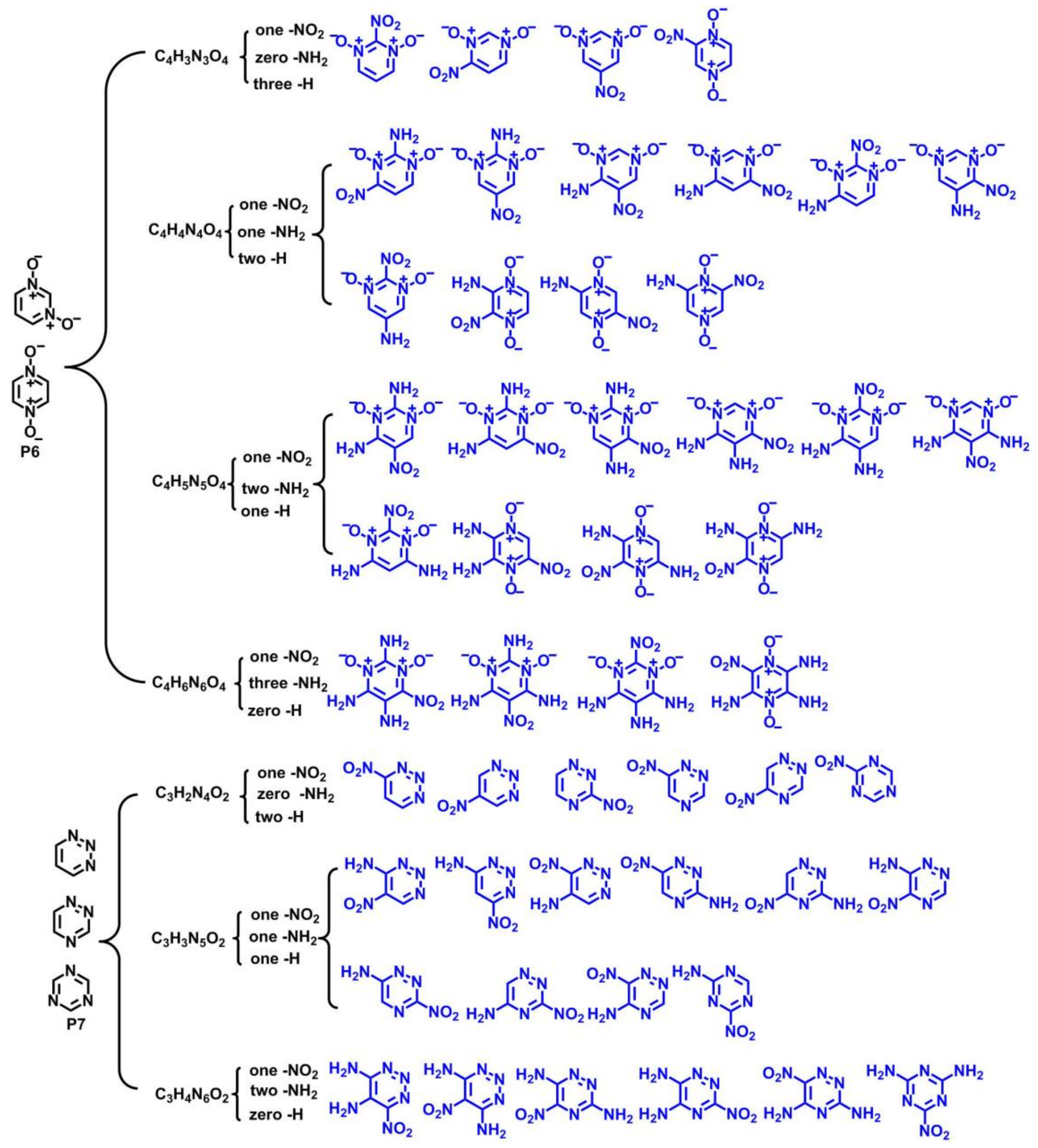

Supplementary Figure 7. Screened molecules by a combination between P6-P7 parent rings and three substituent groups (-NH_2, -NO_2 and -H) with Oxygen Balance constrain.

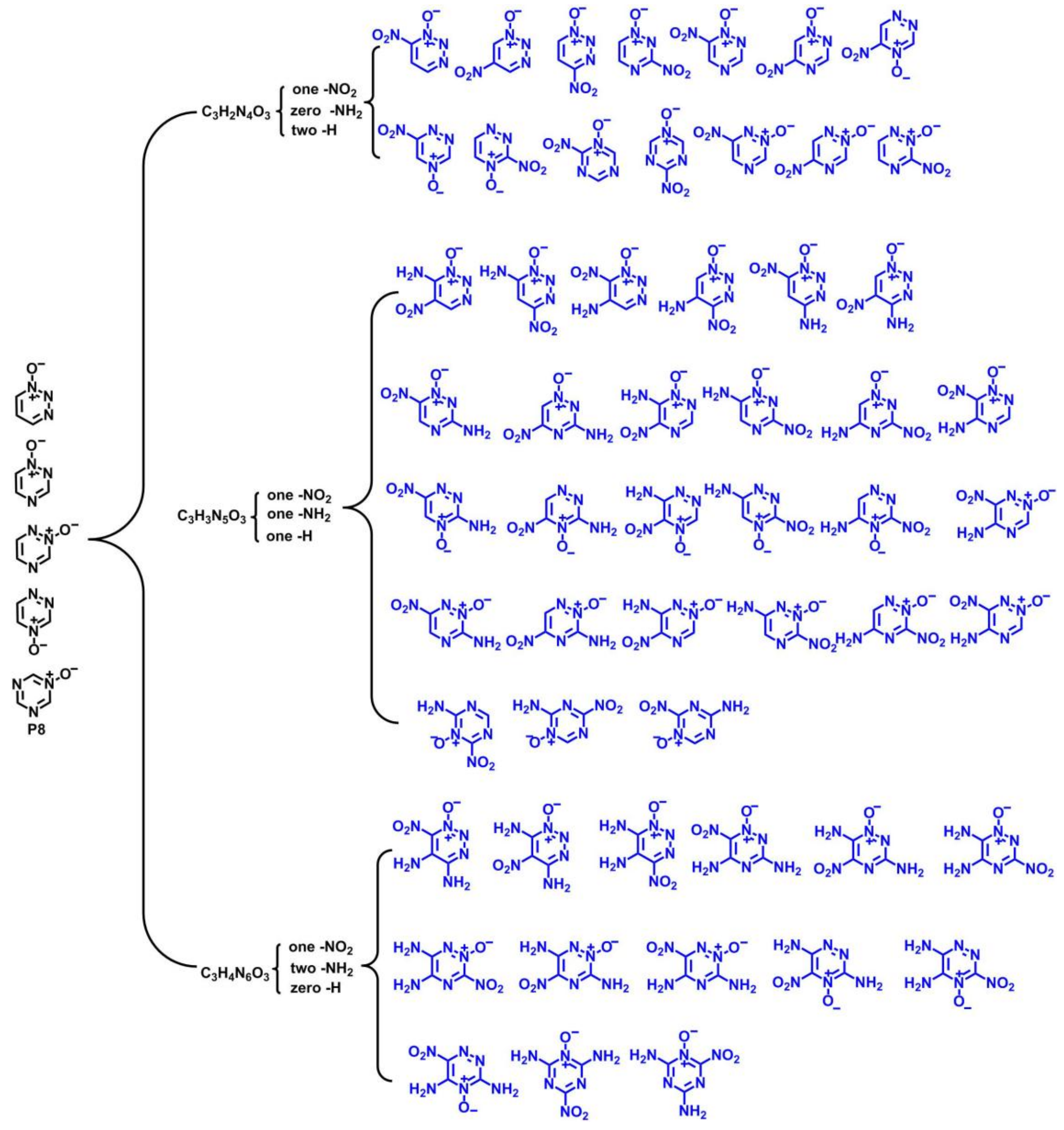

Supplementary Figure 8. Screened molecules by a combination between P8 parent ring and three substituent groups (-NH_2, -NO_2 and -H) with Oxygen Balance constrain.

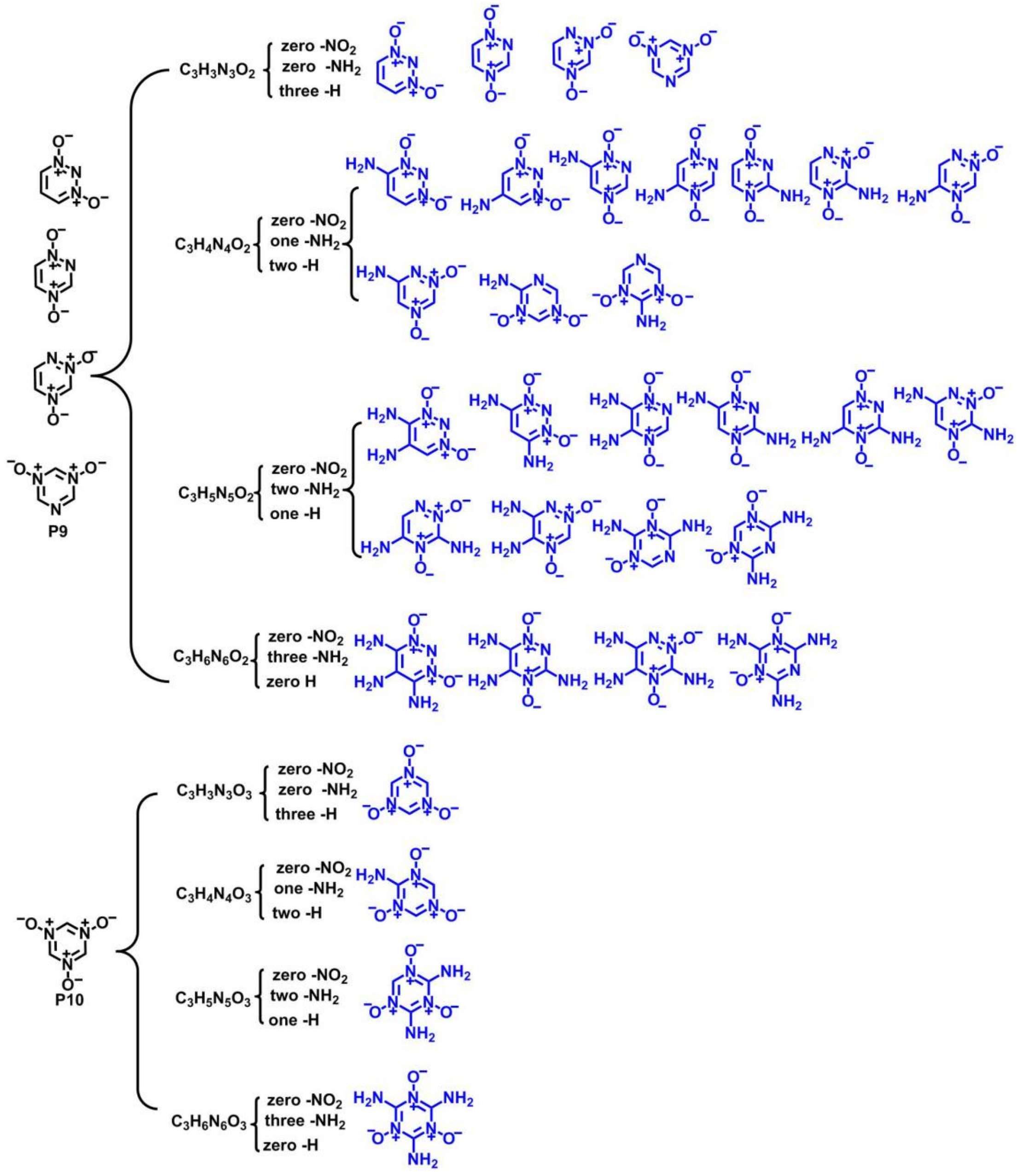

Supplementary Figure 9. Screened molecules by a combination between P9-P10 parent rings and three substituent groups (-NH_2, -NO_2 and -H) with Oxygen Balance constrain.

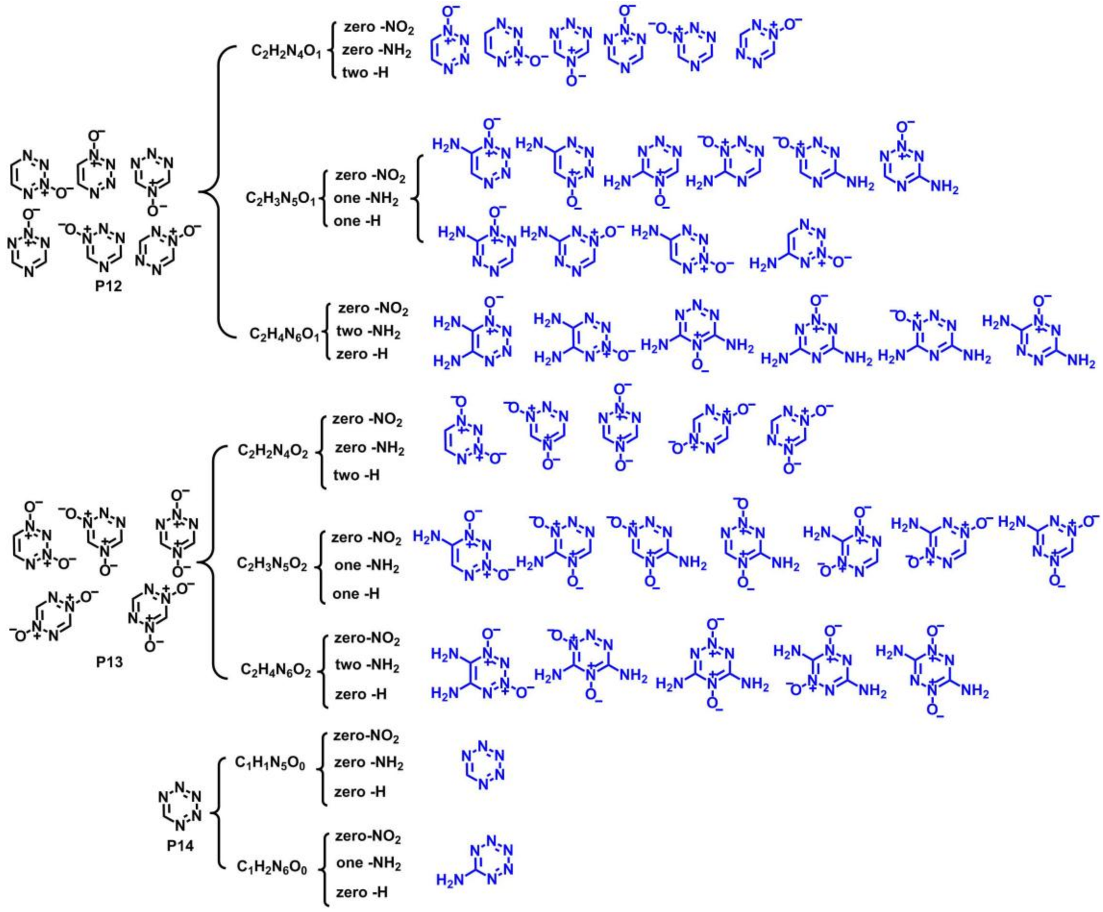

Supplementary Figure 10. Screened molecules by a combination between P12-P14 parent rings and three substituent groups (-NH_2, -NO_2 and -H) with Oxygen Balance constrain.

As shown in Supplementary Figs 4-10, under the constrain of CO_2-based Oxygen Balance in the range of -80% ~ -40%, the final screened molecules by a combination between P1-P14 parent rings and three substituent groups (-NH2, -NO2 and -H) are 402.

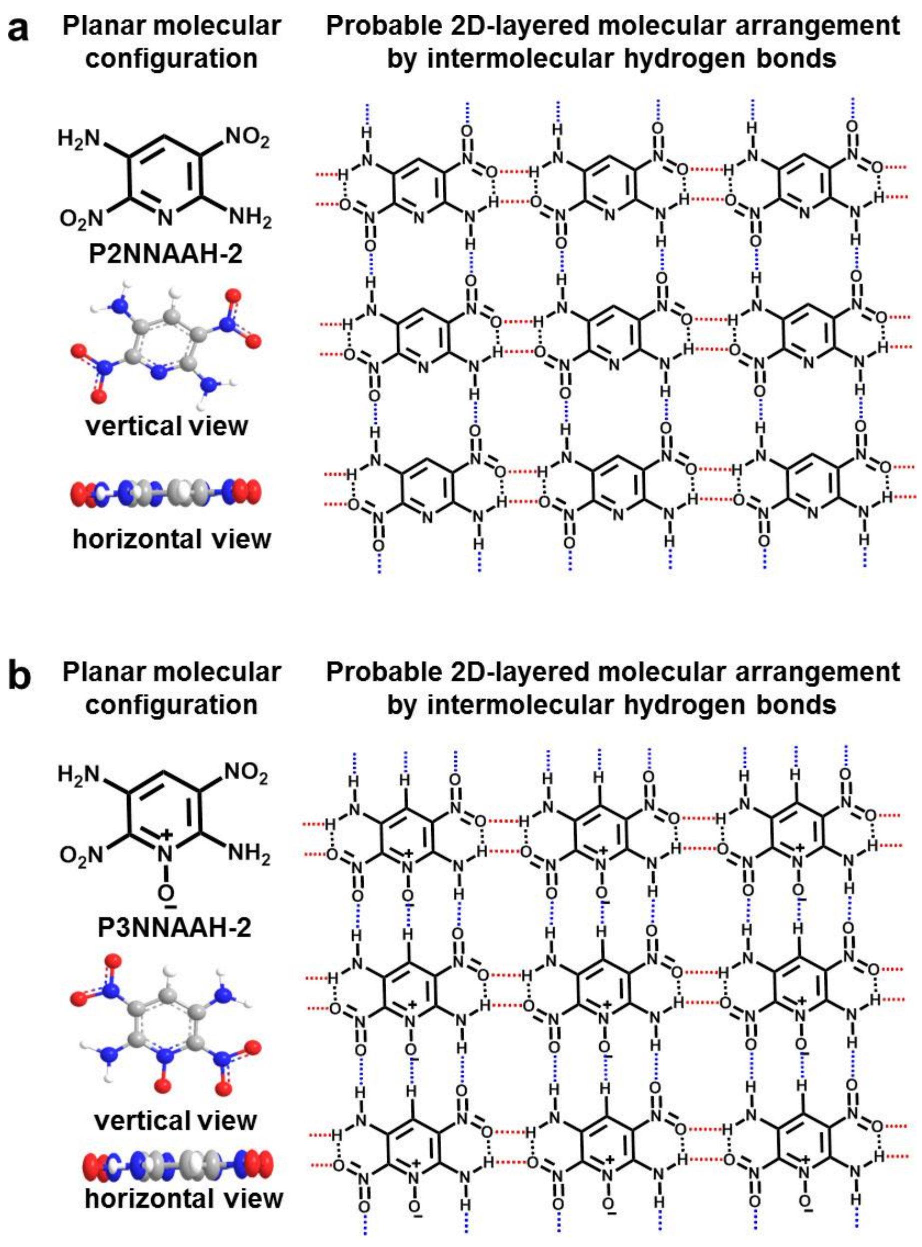

Supplementary Figure 11. Probable 2D-layered structure of P2NNAAH-2 and P3NNAAH-2. **a** Molecular configuration and probable 2D-layered molecular arrangement by some suitable intermolecular hydrogen bonds of P2NNAAH-2. **b** Molecular configuration and probable 2D-layered molecular arrangement by some suitable intermolecular hydrogen bonds of P3NNAAH-2.

Because these molecules have planar molecular configuration and can also self-assemble into 2D molecular sheets by some suitable intermolecular hydrogen bonds, their graphite-like crystal structures could be expected by further π-π stacking between 2D molecular sheets.

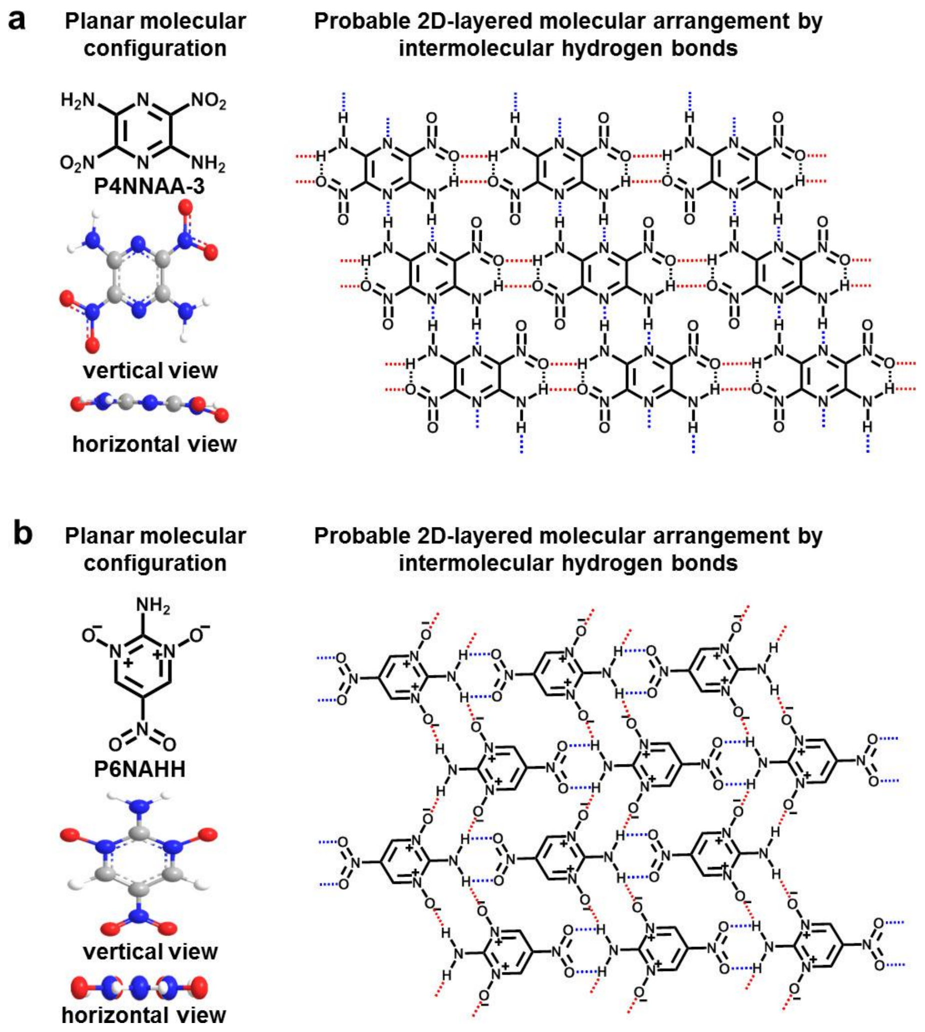

Supplementary Figure 12. Probable 2D-layered structure of P4NNAA-3 and P6NAHH. **a** Molecular configuration and probable 2D-layered molecular arrangement by some suitable intermolecular hydrogen bonds of P4NNAA-3. **b** Molecular configuration and probable 2D-layered molecular arrangement by some suitable intermolecular hydrogen bonds of P6NAHH.

Because these molecules have planar molecular configuration and can also self-assemble into 2D molecular sheets by some suitable intermolecular hydrogen bonds, their graphite-like crystal structures could be expected by further π-π stacking between 2D molecular sheets.

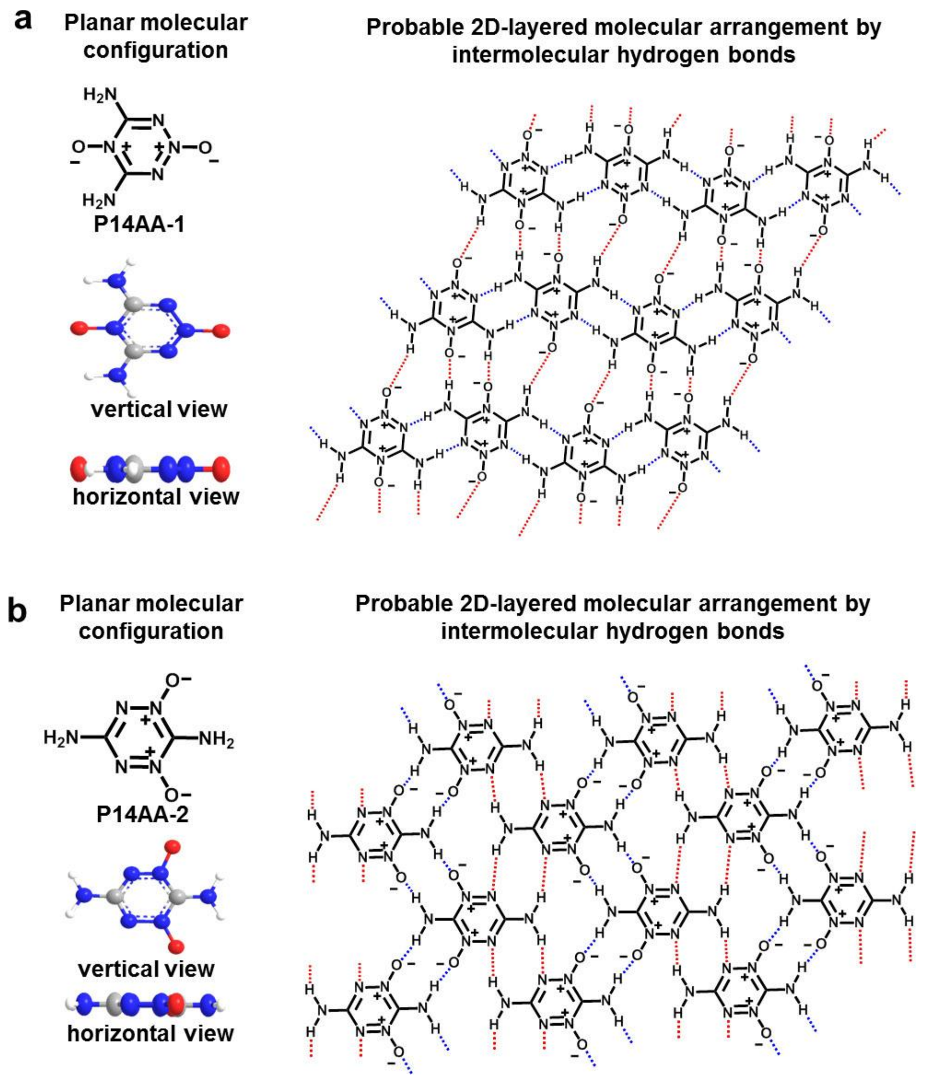

Supplementary Figure 13. Probable 2D-layered structure of P14AA-1 and P14AA-2. **a** Molecular configuration and probable 2D-layered molecular arrangement by some suitable intermolecular hydrogen bonds of P14AA-1. **b** Molecular configuration and probable 2D-layered molecular arrangement by some suitable intermolecular hydrogen bonds of P14AA-2.

Because these molecules have planar molecular configuration and can also self-assemble into 2D molecular sheets by some suitable intermolecular hydrogen bonds, their graphite-like crystal structures could be expected by further π-π stacking between 2D molecular sheets.

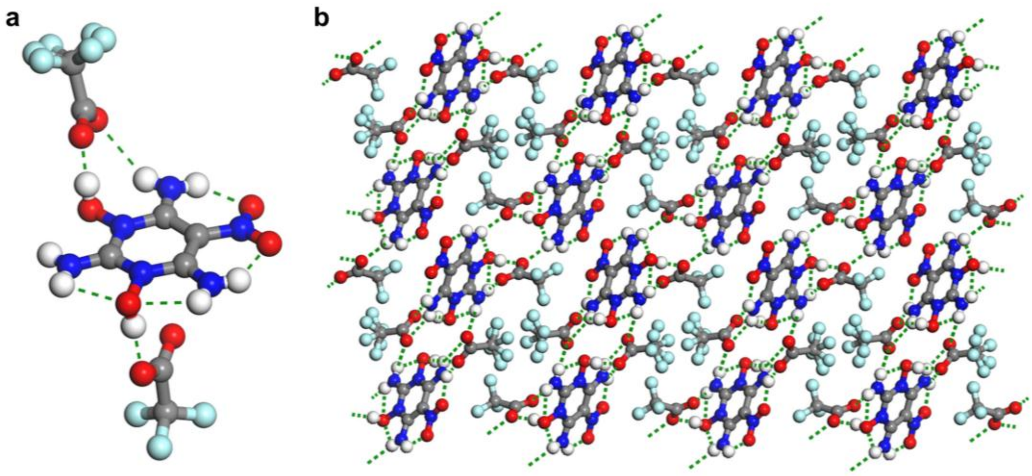

Supplementary Figure 14. **X-ray crystal structure of trifluoroacetate salt of ICM-102**. **a** Hydrogen bonds between protonated ICM-102 and two CF_3COO^- in the trifluoroacetate salt of ICM-102. **b** 3D crystal structure of trifluoroacetate salt of ICM-102 from the view of *b* axis.

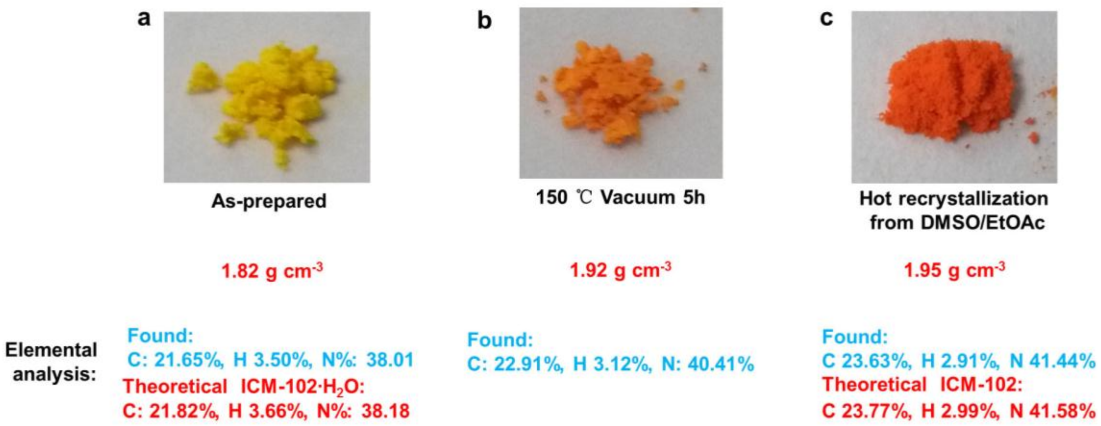

Supplementary Figure 15. **Removing crystal waters of ICM-102 monohydrate by heating treatment**. **a** Optical images, measured density and elemental analysis of as-prepared ICM-102. **b** Optical images, measured density and elemental analysis of ICM-102 dried under vacuum (5 torr) at 150 ^{o}C for 5 hours. **c** Optical images, measured density and elemental analysis of ICM-102 recrystallized from hot DMSO/EtOAc at 130 ^{o}C.

Compounds	Intermolecular hydrogen bond (distance of N-H···O)		Distance between two molecular layers	
	List of different distances (Å)	Average (Å)	List of different distances (Å)	Average (Å)
ICM-102	2.124, 2.152, 2.369, 2.317, 2.048, 1.995,1.977	2.143	3.189, 3.199	3.194
TATB	2.371, 2.348, 2.381, 2.392, 2.396, 2.239	2.355	3.139	3.139
FOX-7	2.446, 2.341, 2.291, 2.145, 2.143	2.273	3.095	3.095
LLM-105	2.509, 2.363, 2.093, 2.046	2.253	2.896	2.896
NTO	2.517, 2.187, 2.011, 2.036	2.188	3.035, 2.983, 2.951, 2.894	2.966

a

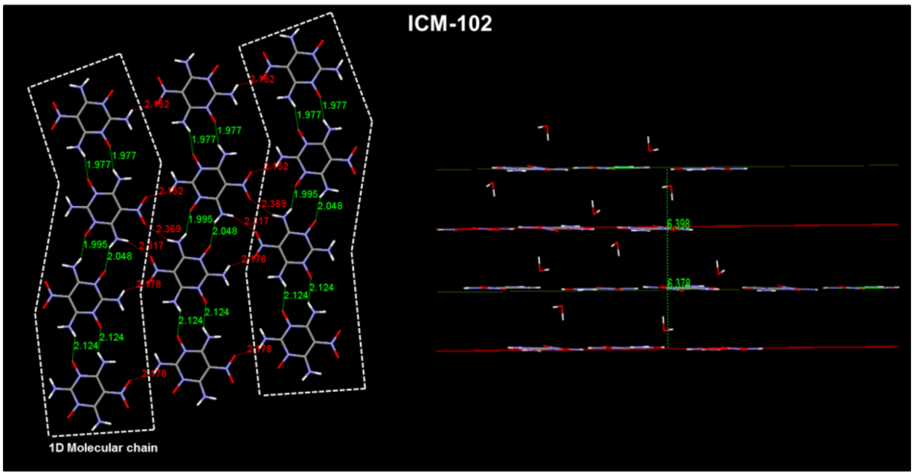

b

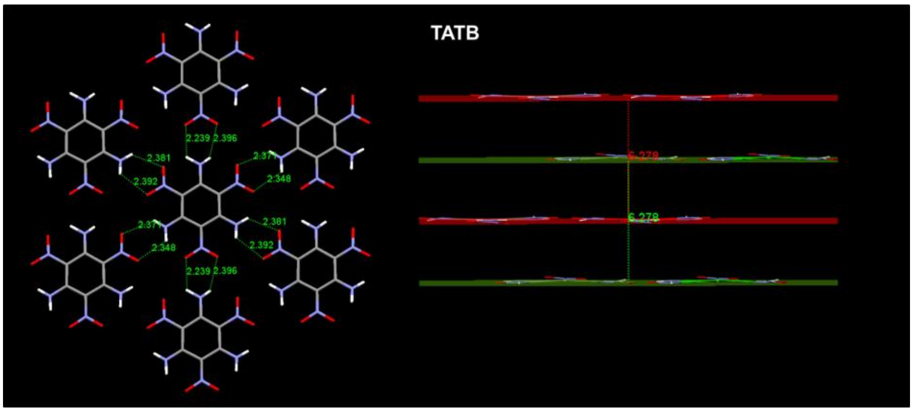

c

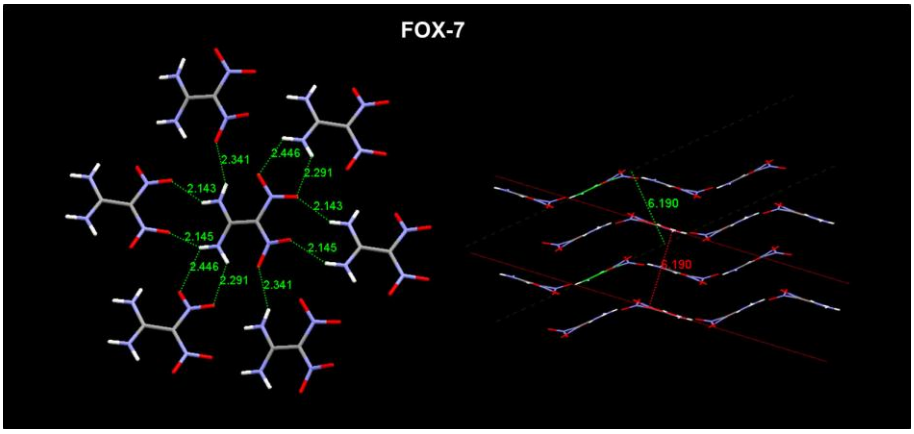

d

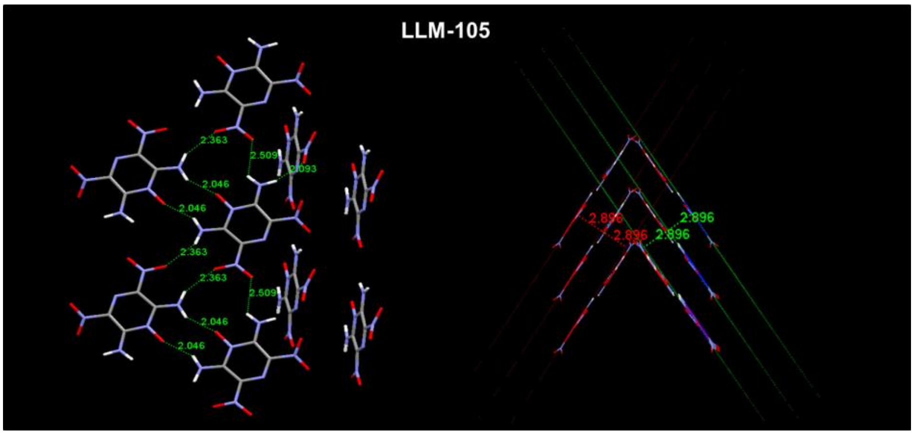

e

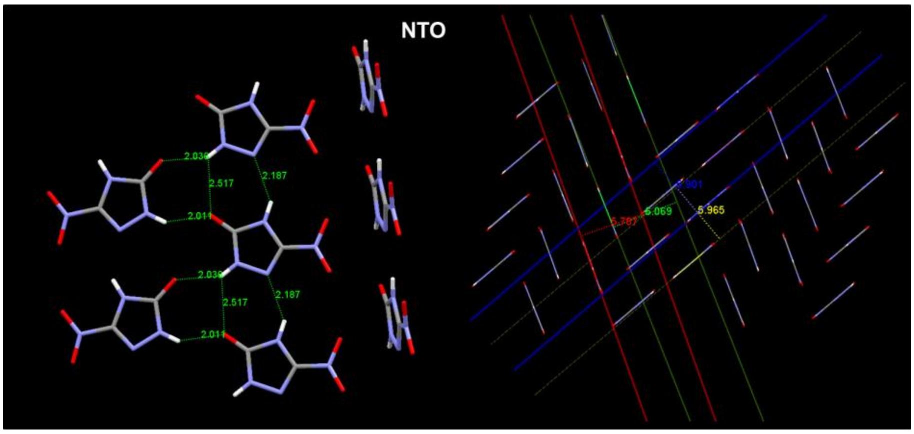

f

Supplementary Figure 16. Intermolecular interactions of ICM-102 and other layered explosives. **a** Comparison of intermolecular hydrogen bond distance and interlamellar spacing between ICM-102 and other layered explosives (TATB, FOX-7, LLM-105 and NTO). **b~f** Intermolecular hydrogen bond distance and interlamellar spacing of ICM-102, TATB, FOX-7, LLM-105 and NTO.

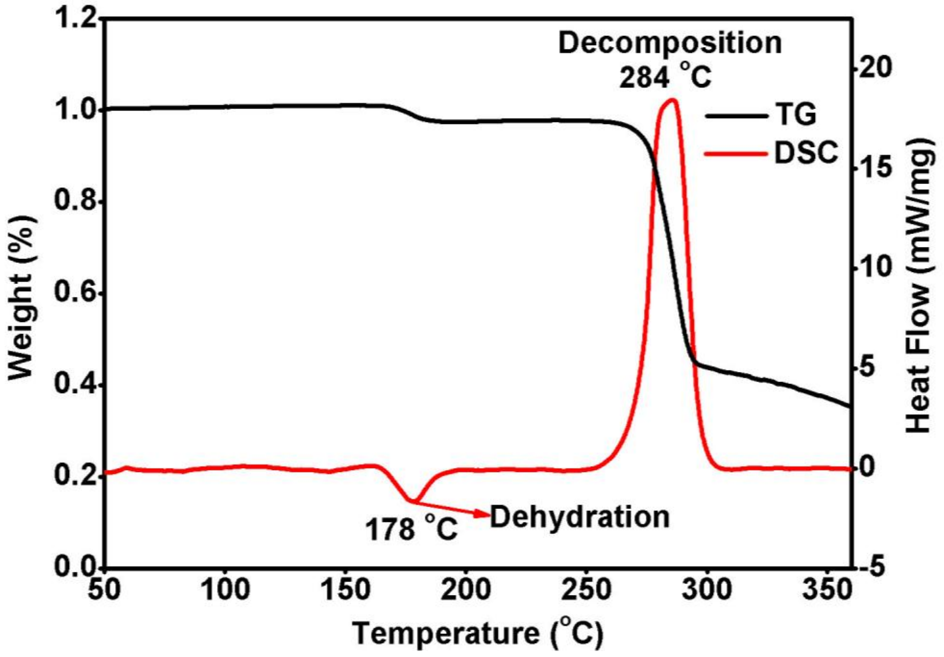

Supplementary Figure 17. **Thermodynamic property of ICM-102 monohydrate**. DSC and TG curves of as-prepared ICM-102 (namely ICM-102 monohydrate).

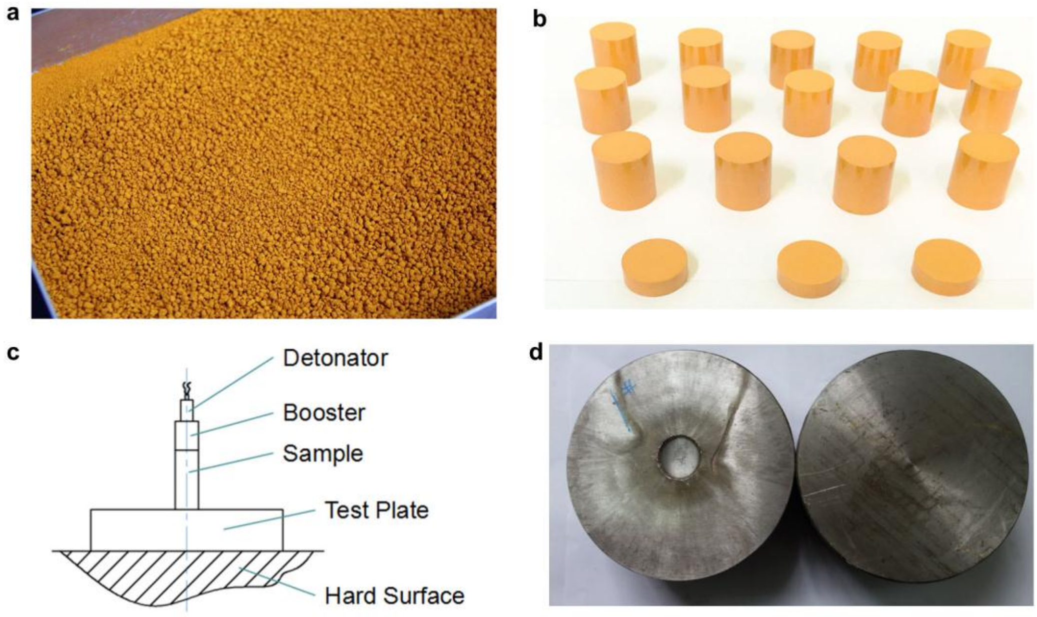

Supplementary Figure 18. Plate-dent test of ICM-102. **a** Photograph of 200 grams of ICM-102. **b** Cylindrical grain of ICM-102 with the a standard of φ 20 × 20 mm. **c** Illustration of the plate-dent test setup. **d** Photograph of steel anvil (φ 100 × 40 mm) before (right) and after (left) the initiability of ICM-102.

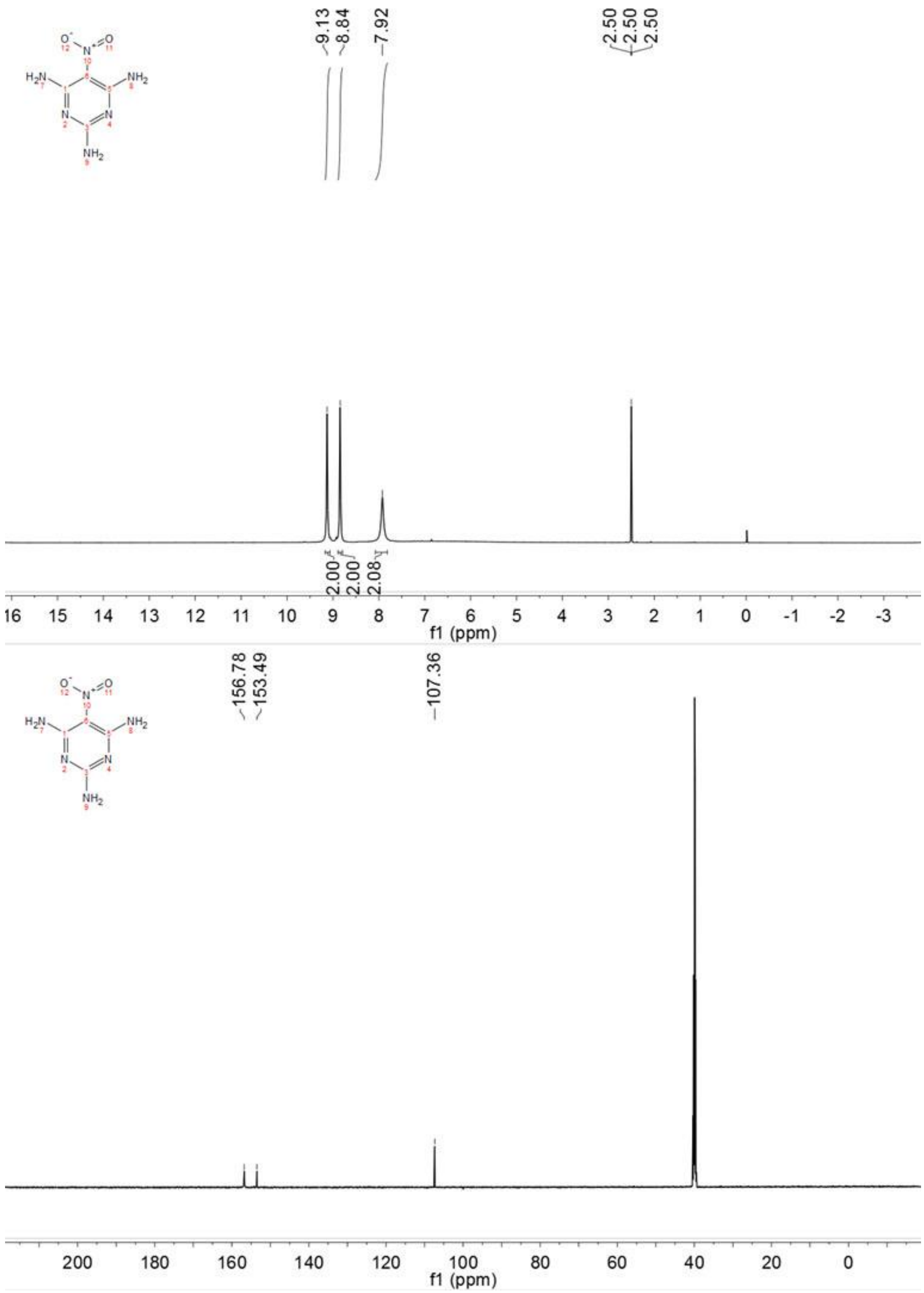

Supplementary Figure 19. ^{1}**H NMR and** ^{13}**C NMR of 2,4,6-triamino-5-nitropyrimidine**.

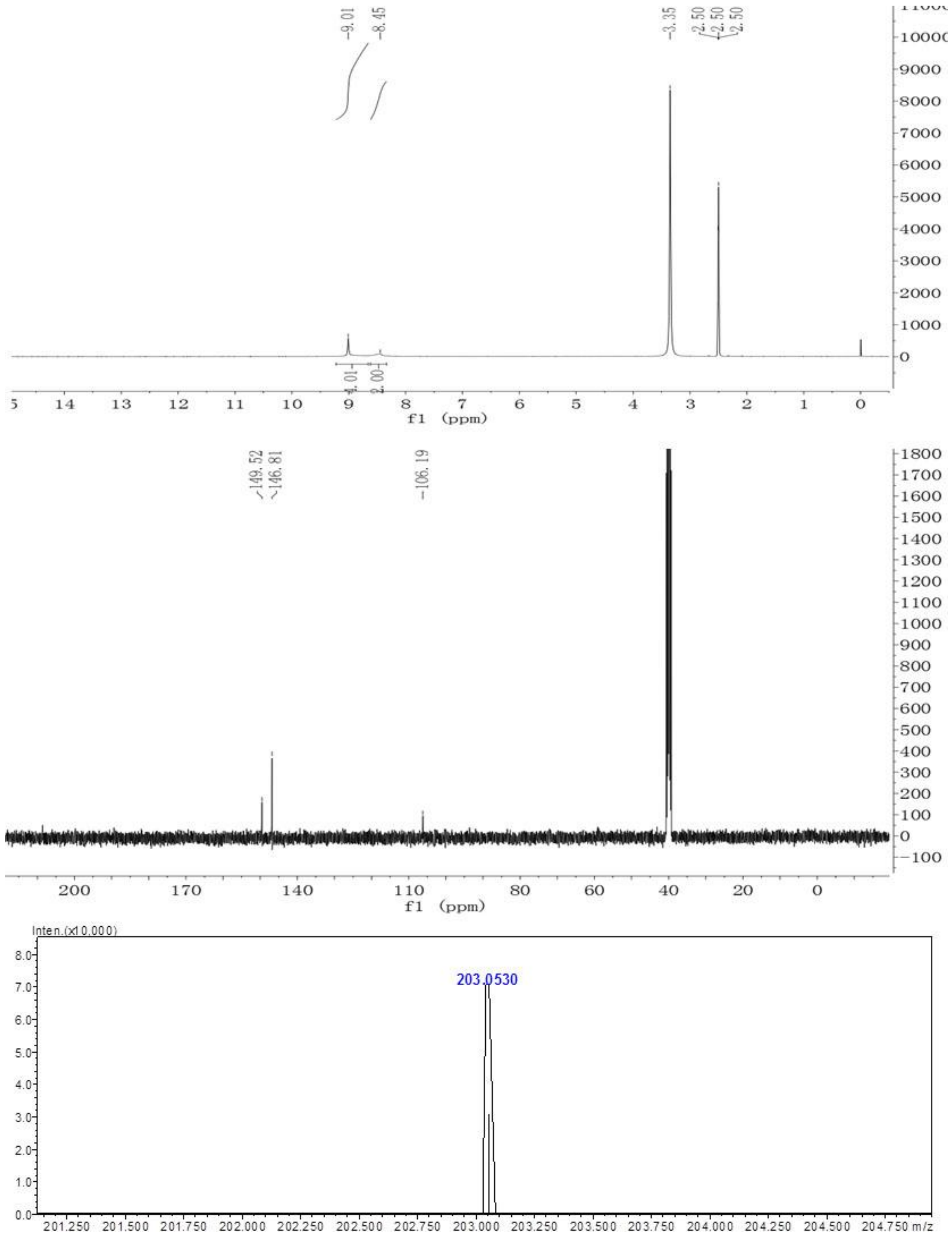

Supplementary Figure 20. ^{1}H NMR, ^{13}C NMR and ESI-HRMS of ICM-102.

Supplementary Table 1. Crystallographic data for trifluoroacetate salt of ICM-102. Single crystal X-ray diffraction data was collected on an Oxford Xcalibur diffratometer with Mo-K_{α} monochromated radiation (λ = 0.71073 Å). The crystal structures were solved by direct methods. The structures were refined on F^2 by full-matrix least-squares methods using the SHELXTL script package.[1] All non-hydrogen atoms were refined anisotropoically.

CCDC	1831055
Formula	$C_4H_8N_6O_4{\cdot}2(C_2F_3O_2^-)$
Mr	430.20
Crystal system	monoclinic
Space group	P 21/c
a [Å]	12.659(3)
b [Å]	5.8144(15)
c [Å]	20.117(5)
α [^{o}]	90
β [^{o}]	92.309(6)
γ [^{o}]	90
V [$Å^3$]	1479.5(6)
Z	4
T(K)	173
ρ[g cm^{-3}]	1.931
Mu [mm^{-1}]	0.212
F(000)	864
θ [^{o}]	1.610 to 25.026
index range	-15≤ h ≤ 14
	-6≤ k ≤ 6
	-23≤ l ≤ 23
reflections collected	10310
independent reflections	2594 [R_{int} = 0.0874, R_{sigma} = 0.0838]
data/restraints/paraneters	2594/38/297
GOF on F^2	1.000
*R*1 [I>2σ(I)]	0.0602
*wR*2 [I>2σ(I)]	0.1543
*R*1(all data)	0.1117
*wR*2(all data)	0.1833
largest diff. peak and hole [e $Å^{-3}$]	0.56/-0.34

Supplementary Table 2. Crystallographic data of ICM-102 monohydrate. Single crystal X-ray diffraction data was collected on an Oxford Xcalibur diffratometer with Mo-K_{α} monochromated radiation (λ = 0.71073 Å). The crystal structures were solved by direct methods. The structures were refined on F^2 by full-matrix least-squares methods using the SHELXTL script package.[1] All non-hydrogen atoms were refined anisotropoically.

CCDC	1816963
Formula	$C_4H_6N_6O_4{\bullet}H_2O$
Mr	220.16
Crystal system	monoclinic
Space group	P 21/c
a [Å]	10.714(2)
b [Å]	8.8863(18)
c [Å]	18.946(4)
α [^{o}]	90
β [^{o}]	118.50(1)
γ [^{o}]	90
V [$Å^3$]	1585.2(6)
Z	8
T(K)	173
ρ[g cm^{-3}]	1.845
Mu [mm^{-1}]	0.167
F(000)	912.0
θ [^{o}]	2.163 to 27.477
index range	$-13 \le h \le 13$
	$-9 \le k \le 11$
	$-24 \le l \le 24$
reflections collected	10126
independent reflections	3526 [R_{int} = 0.0796, R_{sigma} = 0.0992]
data/restraints/paraneters	3526/3/277
GOF on F^2	1.029
*R*1 [I>2σ(I)]	0.0671
*wR*2 [I>2σ(I)]	0.1672
*R*1(all data)	0.1272
*wR*2(all data)	0.2179
largest diff. peak and hole [e $Å^{-3}$]	0.48/-0.55

Supplementary Table 3. **Non-isothermal kinetic parameters of ICM-102**.

	E (kJ/mol)	ln (A)	r
Kissinger	448.7	97.4	0.983
Ozawa	435.5	---	0.984
Average	442.5	---	---

The non-isothermal kinetic parameters of ICM-102 were calculated by Kissinger method and Ozawa method,[2-3] whose equations are as follows:

$$\ln\left(\frac{\beta}{T_{\mathrm{p}}^{2}}\right) = \ln\left(\frac{AR}{E}\right) - \frac{E}{RT_{p}} \qquad (1)$$

$$\log\beta + \frac{0.4567E}{RT_{P}} = C \qquad (2)$$

As shown in Supplementary Table 3, the apparent activation energies (*E*) obtained by the two methods were agree well with each other, and the r (linear correlation coefficient) were both close to 1, indicating that the calculated results are reliable.

Supplementary Table 4. **Evaluation of thermostability of ICM-102**. Onset and peak temperature from DSC curves and extrapolated values of ICM-102 and calculated T_{SADT} and T_b of ICM-102.

heating rate	T_e(℃)	T_p(℃)
5	264.6	280.5
10	271.9	283.7
15	274.4	286.7
0	252.5	277.1

T_{SADT}	T_b
252.5℃	257.7℃

The onset (T_e) and peak temperature (T_p) at different heating rates in DSC cures were listed in Supplementary Table 4. Fitting these data with the function form (3), the extrapolated values when heating rate was 0 can be obtained and were listed in the last row of Supplementary Table 4, which can be labeled as $T_{(0,e)}$ and $T_{(0,P)}$. These values can be used to calculate self-accelerating decomposition temperature (T_{SADT}) and critical temperature of thermal explosion (T_b) combined with apparent activation energy.[4]

$$T_{(0,\ \mathrm{eorp})i} = T_{(\infty,\ \mathrm{eorp})i} + b\beta_i + c\beta_i^2 \qquad (3)$$

$$i = 1\text{-}3$$

The relationships between T_{SADT}, T_b and T_{e0} can be described by equation (4) and equation (5)[5-6]. And the results of T_{SADT} and T_b were 252.5℃ and 257.7℃ when using E calculated by Kissinger method. The two values are high, which means a good thermal stability for ICM-102 during storage and formulation operations.

$$T_{SADT} = T_{e0} \qquad (4)$$

$$T_b = \frac{E_0 - \sqrt{E_0^2 - 4E_0RT_{e0}}}{2R} \qquad (5)$$

Supplementary Table 5. **Solubility of ICM-102 in various solvents at 25 ^{o}C**. For evaluating the solubility in common organic solvents, eight common organic solvents were tested.

Solvent	Water	DMSO	DMF	Ethanol	Methanol
Solubility (mg/100 mL solvent)	220	20	10	<8	<8
Solvent	MeCN	Acetone	DCM	EtOAc	
Solubility (mg/100mL solvent)	<8	<8	<8	<8	

Supplementary Note 1. Rapid screening of six-member aromatic molecules. Permutation and combination between 14 six-member aromatic rings (P1~P14) and three substituent groups (-NH_2, -NO_2 and -H).

It is well known that the organic aromatic explosives can be seen as a combination of aromatic parent rings and substituent groups. To realize rapid and high-throughput screening aromatic explosives, enumerating the possible permutation and combinations between aromatic parent rings and substituent groups would be an efficient way. As shown in Fig. 3 in main text, we choose 14 six-member aromatic rings (P1~P14) and three substituent groups (namely -NH_2, -NO_2 and -H). Here, the self-compiled java scripts help to achieve high-throughput screening new six-member aromatic or hetero-aromatic explosives. Take P2 parent ring (namely C5N1) as an example, we present the establishment of java screening script:

For P2 parent ring, it has 5 carbon atoms and 1 nitrogen atom. Because there are no carbon atom in the selected three substituent groups (-NH_2, -NO_2 and -H), the value of a in molecular formula of $C_aH_bN_cO_d$ is 5. If the numbers of three substituent groups of -NH_2, -NO_2 and -H are x, y, z, respectively, there are some relationships between x, y, z and a, b, c, as shown below:

(1) The total number of substituent groups should be fixed to 5 because only five sites (5 carbon atoms) can be appended by substituting groups at most, which leads to x+y+z = 5, and 0<=x<=6, 0<=y<=6, 0<=z<=6.

(2) According to the rule of nitrogen atomic conservation in a molecule, an equation is established: x+y+1 = c.

(3) According to the rule of hydrogen atomic conservation in a molecule, an equation is established: 2x+z= b.

(4) According to the rule of oxygen atomic conservation in a molecule, an equation is established: 2y = d.

(5) Further, the values of b, c, d are defined to lower than 20. Oxygen atom only shows in pair (in –NO_2), so the value of d should be even number, which is described in java language as “d%2==0”.

Combined with above five conditions, we got some primary screening rules:

b-c+d=4

0<=c-0.5*d-1<=6

0<=b-2*c+d+2<=6

0<=0.5*d<=6 and d%2==0.

The final java script is listed as shown below:

```java
package demo;
public class C5N1_java_source_code {
    public static void main(String[] args) {
        int count = 0;
        int a = 5;
        for (int b=0; b<=20; b++) {
            for (int c=0; c<=20; c++) {
                for (int d=0; d<=20; d++) {
                    if ((b-c+d)==4
                        && (0<=c-0.5*d-1)
                        && (c-0.5*d-1<=6)
                        && (0<=b-2*c+d+2)
                        && (b-2*c+d+2<=6)
                        && (0<=0.5*d)
                        && (0.5*d<=6)
                        && (d%2==0)) {
                        System.out.print(" C" + a + " H" + b + " N"+ c +" O"+ d);
                        System.out.println();
                        count++;
                    }
                }
            }
        }
        System.out.println(count);
    }
}
```

The output formulas were:

C5 H0 N6 O10

C5 H1 N5 O8

C5 H2 N4 O6

C5 H2 N6 O8

C5 H3 N3 O4

C5 H3 N5 O6

C5 H4 N2 O2

C5 H4 N4 O4

C5 H4 N6 O6

C5 H5 N1 O0

C5 H5 N3 O2

C5 H5 N5 O4

C5 H6 N2 O0

C5 H6 N4 O2

C5 H6 N6 O4

C5 H7 N3 O0

C5 H7 N5 O2

C5 H8 N4 O0

C5 H8 N6 O2

C5 H9 N5 O0

C5 H10 N6 O0

There are 21 formulas in total for P2 parent ring with three substituent groups ($-NH_2$, $-NO_2$ and -H).

The similar procedures with minor adjustments are needed for the permutation and combinations between parent rings (P1, P3~P14) and three substituent groups ($-NH_2$, $-NO_2$ and -H). The number of screened formulas is 176 and listed as follow:

Supplementary Table 6. Screened formulas by a combination between P1-P2 parent rings and three substituent groups ($-NH_2$, $-NO_2$ and -H).

Parent-ring sequence	Substituent-group sequence			Selected molecular formula
	$-NO_2$ (N) number	$-NH_2$ (A) number	-H (H) number	
P1	6	0	0	$C_6H_0N_6O_{12}$
	5	0	1	$C_6H_1N_5O_{10}$
	4	0	2	$C_6H_2N_4O_8$
	5	1	0	$C_6H_2N_6O_{10}$
	3	0	3	$C_6H_3N_3O_6$
	4	1	1	$C_6H_3N_5O_8$
	2	0	4	$C_6H_4N_2O_4$
	3	1	2	$C_6H_4N_4O_6$
	4	2	0	$C_6H_4N_6O_8$
	1	0	5	$C_6H_5N_1O_2$
	2	1	3	$C_6H_5N_3O_4$
	3	2	1	$C_6H_5N_5O_6$
	0	0	6	$C_6H_6N_0O_0$
	1	1	4	$C_6H_6N_2O_2$
	2	2	2	$C_6H_6N_4O_4$
	3	3	0	$C_6H_6N_6O_6$
	0	1	5	$C_6H_7N_1O_0$
	1	2	3	$C_6H_7N_3O_2$
	2	3	1	$C_6H_7N_5O_4$
	0	2	4	$C_6H_8N_2O_0$
	1	3	2	$C_6H_8N_4O_2$
	2	4	0	$C_6H_8N_6O_4$
	0	3	3	$C_6H_9N_3O_0$
	1	4	1	$C_6H_9N_5O_2$
	0	4	2	$C_6H_{10}N_4O_0$
	1	5	0	$C_6H_{10}N_6O_2$
	0	5	1	$C_6H_{11}N_5O0$
	0	6	0	$C_6H_{12}N_6O_0$

Parent-ring sequence	Substituent-group sequence			Selected molecular formula
	$-NO_2$ (N) number	$-NH_2$ (A) number	-H (H) number	
P2	5	0	0	$C_5H_0N_6O_{10}$
	4	0	1	$C_5H_1N_5O_8$
	3	0	2	$C_5H_2N_4O_6$
	4	1	0	$C_5H_2N_6O_8$
	2	0	3	$C_5H_3N_3O_4$
	3	1	1	$C_5H_3N_5O_6$
	1	0	4	$C_5H_4N_2O_2$
	2	1	2	$C_5H_4N_4O_4$
	3	2	0	$C_5H_4N_6O_6$
	0	0	5	$C_5H_5N_1O_0$
	1	1	3	$C_5H_5N_3O_2$
	2	2	1	$C_5H_5N_5O_4$
	0	1	4	$C_5H_6N_2O_0$
	1	2	2	$C_5H_6N_4O_2$
	2	3	0	$C_5H_6N_6O_4$
	0	2	3	$C_5H_7N_3O_0$
	1	3	1	$C_5H_7N_5O_2$
	0	3	2	$C_5H_8N_4O_0$
	1	4	0	$C_5H_8N_6O_2$
	0	4	1	$C_5H_9N_5O_0$
	0	5	0	$C_5H_{10}N_6O_0$

Supplementary Table 7. Screened formulas by a combination between P3-P6 parent rings and three substituent groups (-NH_2, -NO_2 and -H).

Parent-ring sequence	Substituent-group sequence			Selected molecular formula
	-NO_2 (N) number	-NH_2 (A) number	-H (H) number	
P3	5	0	0	$C_5H_0N_6O_{11}$
	4	0	1	$C_5H_1N_5O_9$
	3	0	2	$C_5H_2N_4O_7$
	4	1	0	$C_5H_2N_6O_9$
	2	0	3	$C_5H_3N_3O_5$
	3	1	1	$C_5H_3N_5O_7$
	1	0	4	$C_5H_4N_2O_3$
	2	1	2	$C_5H_4N_4O_5$
	3	2	0	$C_5H_4N_6O_7$
	0	0	5	$C_5H_5N_1O_1$
	1	1	3	$C_5H_5N_3O_3$
	2	2	1	$C_5H_5N_5O_5$
	0	1	4	$C_5H_6N_2O_1$
	1	2	2	$C_5H_6N_4O_3$
	2	3	0	$C_5H_6N_6O_5$
	0	2	3	$C_5H_7N_3O_1$
	1	3	1	$C_5H_7N_5O_3$
	0	3	2	$C_5H_8N_4O_1$
	1	4	0	$C_5H_8N_6O_3$
	0	4	1	$C_5H_9N_5O_1$
	0	5	0	$C_5H_{10}N_6O_1$

Parent-ring sequence	Substituent-group sequence			Selected molecular formula
	-NO_2 (N) number	-NH_2 (A) number	-H (H) number	
P4	4	0	0	$C_4H_0N_6O_8$
	3	0	1	$C_4H_1N_5O_6$
	2	0	2	$C_4H_2N_4O_4$
	3	1	0	$C_4H_2N_6O_6$
	1	0	3	$C_4H_3N_3O_2$
	2	1	1	$C_4H_3N_5O_4$
	0	0	4	$C_4H_4N_2O_0$
	1	1	2	$C_4H_4N_4O_2$
	2	2	0	$C_4H_4N_6O_4$
	0	1	3	$C_4H_5N_3O_0$
	1	2	1	$C_4H_5N_5O_2$
	0	2	2	$C_4H_6N_4O_0$
	1	3	0	$C_4H_6N_6O_2$
	0	3	1	$C_4H_7N_5O_0$
	0	4	0	$C_4H_8N_6O_0$

Parent-ring sequence	Substituent-group sequence			Selected molecular formula
	-NO_2 (N) number	-NH_2 (A) number	-H (H) number	
P5	4	0	0	$C_4H_0N_6O_9$
	3	0	1	$C_4H_1N_5O_7$
	2	0	2	$C_4H_2N_4O_5$
	3	1	0	$C_4H_2N_6O_7$
	1	0	3	$C_4H_3N_3O_3$
	2	1	1	$C_4H_3N_5O_5$
	0	0	4	$C_4H_4N_2O_1$
	1	1	2	$C_4H_4N_4O_3$
	2	2	0	$C_4H_4N_6O_5$
	0	1	3	$C_4H_5N_3O_1$
	1	2	1	$C_4H_5N_5O_3$
	0	2	2	$C_4H_6N_4O_1$
	1	3	0	$C_4H_6N_6O_3$
	0	3	1	$C_4H_7N_5O_1$
	0	4	0	$C_4H_8N_6O_1$

Parent-ring sequence	Substituent-group sequence			Selected molecular formula
	-NO_2 (N) number	-NH_2 (A) number	-H (H) number	
P6	4	0	0	$C_4H_0N_6O_{10}$
	3	0	1	$C_4H_1N_5O_8$
	2	0	2	$C_4H_2N_4O_6$
	3	1	0	$C_4H_2N_6O_8$
	1	0	3	$C_4H_3N_3O_4$
	2	1	1	$C_4H_3N_5O_6$
	0	0	4	$C_4H_4N_2O_2$
	1	1	2	$C_4H_4N_4O_4$
	2	2	0	$C_4H_4N_6O_6$
	0	1	3	$C_4H_5N_3O_2$
	1	2	1	$C_4H_5N_5O_4$
	0	2	2	$C_4H_6N_4O_2$
	1	3	0	$C_4H_6N_6O_4$
	0	3	1	$C_4H_7N_5O_2$
	0	4	0	$C_4H_8N_6O_2$

Supplementary Table 8. Screened formulas by a combination between P7-P14 parent rings and three substituent groups (-NH$_2$, -NO$_2$ and -H).

Parent-ring sequence	Substituent-group sequence			Selected molecular formula
	-NO$_2$ (N) number	-NH$_2$ (A) number	-H (H) number	
P7	3	0	0	$C_3H_0N_6O_6$
	2	0	1	$C_3H_1N_5O_4$
	1	0	2	$C_3H_2N_4O_2$
	2	1	0	$C_3H_2N_6O_4$
	0	0	3	$C_3H_3N_3O_0$
	1	1	1	$C_3H_3N_5O_2$
	0	1	2	$C_3H_4N_4O_0$
	1	2	0	$C_3H_4N_6O_2$
	0	2	1	$C_3H_5N_5O_0$
	0	3	0	$C_3H_6N_6O_0$

Parent-ring sequence	Substituent-group sequence			Selected molecular formula
	-NO$_2$ (N) number	-NH$_2$ (A) number	-H (H) number	
P8	3	0	0	$C_3H_0N_6O_7$
	2	0	1	$C_3H_1N_5O_5$
	1	0	2	$C_3H_2N_4O_3$
	2	1	0	$C_3H_2N_6O_5$
	0	0	3	$C_3H_3N_3O_1$
	1	1	1	$C_3H_3N_5O_3$
	0	1	2	$C_3H_4N_4O_1$
	1	2	0	$C_3H_4N_6O_3$
	0	2	1	$C_3H_5N_5O_1$
	0	3	0	$C_3H_6N_6O_1$

Parent-ring sequence	Substituent-group sequence			Selected molecular formula
	-NO$_2$ (N) number	-NH$_2$ (A) number	-H (H) number	
P9	3	0	0	$C_3H_0N_6O_8$
	2	0	1	$C_3H_1N_5O_6$
	1	0	2	$C_3H_2N_4O_4$
	2	1	0	$C3H_2N_6O_6$
	0	0	3	$C_3H_3N_3O_2$
	1	1	1	$C_3H_3N_5O_4$
	0	1	2	$C_3H_4N_4O_2$
	1	2	0	$C_3H_4N_6O_4$
	0	2	1	$C_3H_5N_5O_2$
	0	3	0	$C_3H_6N_6O_2$

Parent-ring sequence	Substituent-group sequence			Selected molecular formula
	-NO$_2$ (N) number	-NH$_2$ (A) number	-H (H) number	
P10	3	0	0	$C_3H_0N_6O_9$
	2	0	1	$C_3H_1N_5O_7$
	1	0	2	$C_3H_2N_4O_5$
	2	1	0	$C_3H_2N_6O_7$
	0	0	3	$C_3H_3N_3O_3$
	1	1	1	$C_3H_3N_5O_5$
	0	1	2	$C_3H_4N_4O_3$
	1	2	0	$C_3H_4N_6O_5$
	0	2	1	$C_3H_5N_5O_3$
	0	3	0	$C_3H_6N_6O_3$

Parent-ring sequence	Substituent-group sequence			Selected molecular formula
	-NO$_2$ (N) number	-NH$_2$ (A) number	-H (H) number	
P11	0	0	2	$C_2H_2N_4O_0$
	0	2	0	$C_2H_4N_6O_0$
	2	0	0	$C_2H_0N_6O_4$
	1	1	0	$C_2H_2N_6O_2$
	1	0	1	$C_2H_1N_5O_2$
	0	1	1	$C_2H_3N_5O_0$

Parent-ring sequence	Substituent-group sequence			Selected molecular formula
	-NO$_2$ (N) number	-NH$_2$ (A) number	-H (H) number	
P12	2	0	0	$C_2H_0N_6O_5$
	1	0	1	$C_2H_1N_5O_3$
	0	0	2	$C_2H_2N_4O_1$
	1	1	0	$C_2H_2N_6O_3$
	0	1	1	$C_2H_3N_5O_1$
	0	2	0	$C_2H_4N_6O_1$

Parent-ring sequence	Substituent-group sequence			Selected molecular formula
	-NO$_2$ (N) number	-NH$_2$ (A) number	-H (H) number	
P13	2	0	0	$C_2H_0N_6O_6$
	1	0	1	$C_2H_1N_5O_4$
	0	0	2	$C_2H_2N_4O_2$
	1	1	0	$C_2H_2N_6O_4$
	0	1	1	$C_2H_3N_5O_2$
	0	2	0	$C_2H_4N_6O_2$

Parent-ring sequence	Substituent-group sequence			Selected molecular formula
	-NO$_2$ (N) number	-NH$_2$ (A) number	-H (H) number	
P14	0	0	1	$C_1H_1N_5O_0$
	0	1	0	$C_1H_2N_6O_0$
	1	0	0	$C_1H_0N_6O_2$

Supplementary Note 2. Rapid screening of six-member aromatic molecules with Oxygen Balance constrain. Permutation and combination between 14 six-member aromatic rings (P1~P14) and three substituent groups (-NH_2, -NO_2 and -H) with Oxygen Balance constrain.

Here, we still take P2 parent ring (namely C5N1) as an example. Thus, the same primary screening rules are also needed:

$$b-c+d=4$$
$$0<=c-0.5*d-1<=6$$
$$0<=b-2*c+d+2<=6$$
$$0<=0.5*d<=6 \text{ and } d\%2==0.$$

Based on the analysis of statistical data, we have concluded that a relatively low CO_2-based OB in the range of -80% ~ -40% is desirable for the insensitivity demand of high explosives (Figure 2b). In following screening, an OB rule with a formulation is added as:

$$-0.8< (d-2*a-0.5*b)/(12*a+b+14*c+16*d))*16<-0.4$$

The final java script is listed as shown below:

```
package demo;
public class C5N1_java_source_code {
    public static void main(String[] args) {
        int count = 0;
        int a = 5;
        for (int b=0; b<=6; b++) {
            for (int c=0; c<=6; c++) {
                for (int d=0; d<=6; d++) {
                    if ((-0.8<((d-2*a-0.5*b)/(12*a+b+14*c+16*d))*16
                            && ((d-2*a-0.5*b)/(12*a+b+14*c+16*d))*16<-0.4)
                            && (b-c+d)==4
                            && (0<=c-0.5*d-1)
                            && (c-0.5*d-1<=6)
                            && (0<=b-2*c+d+2)
                            && (b-2*c+d+2<=6)
                            && (0<=0.5*d)
                            && (0.5*d<=6)
                            && (d%2==0)) {
                        System.out.print(" C" + a + " H" + b + " N"+ c +" O"+ d);
```

```
                        System.out.println();
                        count++;
                    }
                }
            }
        }
        System.out.println(count);
    }
}
```

The output formulas were:

C5 H3 N3 O4

C5 H4 N4 O4

C5 H5 N5 O4

C5 H6 N6 O4

There are 4 suitable formulas.

Supplementary Note 3. The theoretical calculations about the heat of formation of ICM-102.

Theoretical calculations were performed by using the Gaussian 09 (Revision D.01) suite of scripts.[7] The geometric optimization and frequency analyses were completed by using the B3LYP functional with the 6-31+G** basis set. Single energy points were calculated at the MP2/6-311++G** level of theory. For all of the compounds, the optimized structures were characterized to be true local energy minima on the potential-energy surface without imaginary frequencies. The isodesmic reaction was carried out to obtain the gas-phase heat of formation of the neutral compound. The gas-phase enthalpies of the building-block molecules were obtained by using the atomization method with the G2 ab initio calculations. Then the remaining task is to determine the solid-state heat of formation for the synthesized compound. The solid-state enthalpy of formation for neutral compound can be estimated by subtracting the heat of sublimation from gas-phase heat of formation. On the basis of the literature,[8-9] the heat of sublimation can be estimated with Trouton's rule according to supplementary equation (6), where T represents either the melting point or the decomposition temperature when no melting occurs prior to decomposition:

$$\Delta H_{sub} = 188\ \mathrm{J\ mol^{-1}\ K^{-1}} \times T \qquad (6)$$

The isodesmic reaction of ICM-102 is as follows:

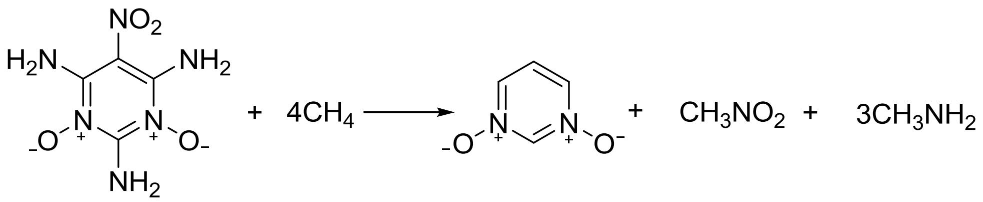

Supplementary Note 4. Theoretical prediction on the crystal density of 7 selected molecules.

The theoretical density was initially determined from the molecular weight (M) divided by the $V_{0.001}$. The $V_{0.001}$ is defined as the volume of that inside the density contour of 0.001 electrons bohr^{-3}, which was obtained from a Monte Carlo integration using MultiWFN program.[10] By introducing the interaction index $\nu\sigma^2_{tot}$, the crystal density of energetic compounds can be corrected according to the following equation (7)[11]:

$$\rho = \beta_1\left(\frac{M}{V_{0.001}}\right) + \beta_2\left(\nu\sigma^2_{tot}\right) + \beta_3 \qquad (7)$$

where β_1, β_2 and β_3 are 1.0462, 0.0021 and 0.1586, respectively.[12]

However, the accurate theoretical prediction of molecular crystal density still remains a great challenge, especially for these compounds with multiple amino and nitro groups. As shown in Supplementary Fig. 21a, the calculated crystal densities are generally lower than those of experimental ones, but their absolute differences are relatively concentrated and fall in the range of 0.14 g cm^{-3} to 0.22 g cm^{-3}. Here, we take the average value (0.18 g cm^{-3}) of their absolute differences as a correction for these calculations (Supplementary Fig. 21a). Therefore, among these 7 selected molecules, P6NAAA will have the highest calculated crystal density (Supplementary Fig. 21b) and is selected as the final research target molecule.

a

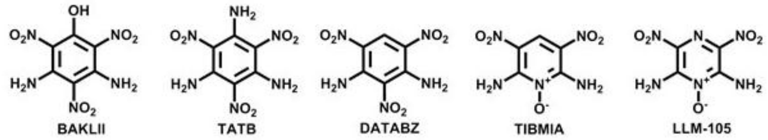

Compounds	M.W. (g mol^{-1})	$V_{0.001}$ (cm^3 mol^{-1})	$\nu\sigma^2_{tot}$ (kcal mol^{-1})	$\rho_{cal.}$ (g cm^{-3})	$\rho_{exp.}$ (g cm^{-3})	$\triangle\rho = \lvert\rho_{exp.}-\rho_{cal.}\rvert$ (g cm^{-3})
BAKLII	259	146.99	32.89	1.75	1.89	0.14
TATB	258	148.75	32.07	1.72	1.94	0.22
DATABZ	243	142.37	31.52	1.69	1.84	0.15
TIBMIA	215	127.80	33.50	1.67	1.88	0.21
LLM-105	216	125.41	45.13	1.74	1.91	0.17
Average						0.18

b

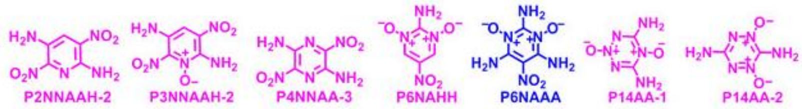

Compounds	M.W. (g mol^{-1})	$V_{0.001}$ (cm^3 mol^{-1})	$\nu\sigma^2_{tot}$ (kcal mol^{-1})	$\rho_{cal.}$ (g cm^{-3})	$\triangle\rho$ (g cm^{-3})	$\rho_{cor.}$ (g cm^{-3})
P2NNAAH-2	199	121.47	48.16	1.66	0.18	1.84
P3NNAAH-2	215	129.96	44.76	1.67	0.18	1.85
P4NNAA-3	200	120.90	39.20	1.65	0.18	1.83
P6NAHH	172	104.16	35.73	1.64	0.18	1.82
P6NAAA	202	118.03	44.86	1.73	0.18	1.91
P14AA-1	144	90.11	65.58	1.65	0.18	1.83
P14AA-2	144	88.76	62.37	1.67	0.18	1.85

Supplementary Figure 21. a Corrected relationship between calculated and experimental crystal densities of some typical six-member aromatic energetic compounds with multiple amino and nitro groups. **b** Calculated crystal density of these 7 selected molecules by further correction.

Supplementary Note 5. Theoretical prediction on the crystal structure of ICM-102.

The single molecule configuration was firstly optimized in Dmol3 (at a level of GGA/PBE and DNP basis set). The crystal structures of ICM-102 was predicted by Polymorph module with a perl script in Materials studio 7.0 software package and the molecule configuration were kept unchanged during the whole process. The space groups used in prediction includes "P21/C", "P-1", "P212121", "C2/C", "PBCA", "PNMA", "PNA21", "P2/C", "PBCN", "P1", "CC", "C2", "PCA21", "P21/m", "P21", "P21212", because they are encompassed 80% known organic molecular crystals.[13] The intermolecular interactions in crystal prediction were described by force field of "Dreiding".[14]

The calculated results show that the crystal structure of ICM-102 with the lowest lattice energy is a P -1 space group with 3D graphite-like layered structure (Supplementary Fig. 22a) and the corresponding 2D molecular sheet is constructed by supramolecular synthon 5 and supramolecular synthon 4 (Supplementary Fig. 22b).

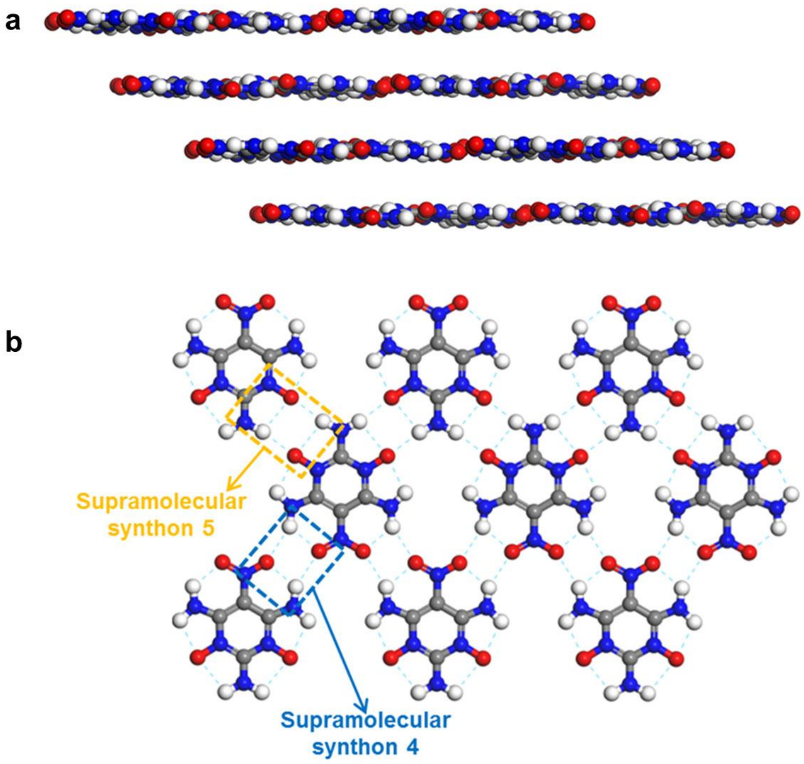

Supplementary Figure 22. a Theoretically predicted 3D graphite-like layered structure of ICM-102 with the lowest lattice energy. **b** Theoretically predicted intermolecular hydrogen bonds in the 2D molecular sheet of ICM-102 with the lowest lattice energy.

Supplementary Note 6. Synthesis of ICM-102 by repeating the reported synthetic methods.

Though three literatures described the synthesis of ICM-102,[15-17] only two papers (reported in 1968 and 2004, respectively) provided the synthetic procedures. After repeating these two papers, we failed to obtain their claimed target molecule, and our new findings are listed as follows:

(1) For the paper published in 1968,[15] the authors claimed that precipitates would be formed after adding water into filtrate and further purification by recrystallization from water. But when we repeated this experiment, we could not obtain the precipitates after adding water into filtrate. In fact, this phenomenon is reasonable because our following studies confirm that the precipitates are trifluoroacetate salt (Supplementary Table 1 and Supplementary Fig. 14) with good solubility in water (see Method part in revised manuscript). We have obtained the crystal of this trifluoroacetate salt and analyzed its crystal structure by single crystal X-ray diffraction, as shown in Supplementary Fig. 14. Therefore, we don't think the synthetic method provided by the paper in 1968 have successfully synthesized the target product ICM-102.

(2) For the paper published in 2004,[17] the authors claimed to modify the synthetic route provided by the paper in 1968, which further indicates that the original synthetic route is unreasonable. In this paper, the authors claimed that precipitates would be formed after adding diethyl ether into the reaction mixture and further purification by washing diethyl ether and boiling propan-2-ol. But when repeating this experiment, we only obtained complex mixtures including large amounts of trifluoroacetate salt, a small amount of desired ICM-102, and some unknown impurities (Supplementary Fig. 23a). Therefore, we don't think the synthetic method provided by the paper in 2004 have successfully synthesized the pure target product ICM-102.

Therefore, we think that the post-treatment operations in previous literatures are unreasonable and the obtained precipitates from highly acidic H_2O_2/CF_3COOH system are primarily the trifluoroacetate salt (Supplementary Fig. 23b). To obtain the neutral ICM-102, an acid-base neutralization step is necessary in post-treatment process.

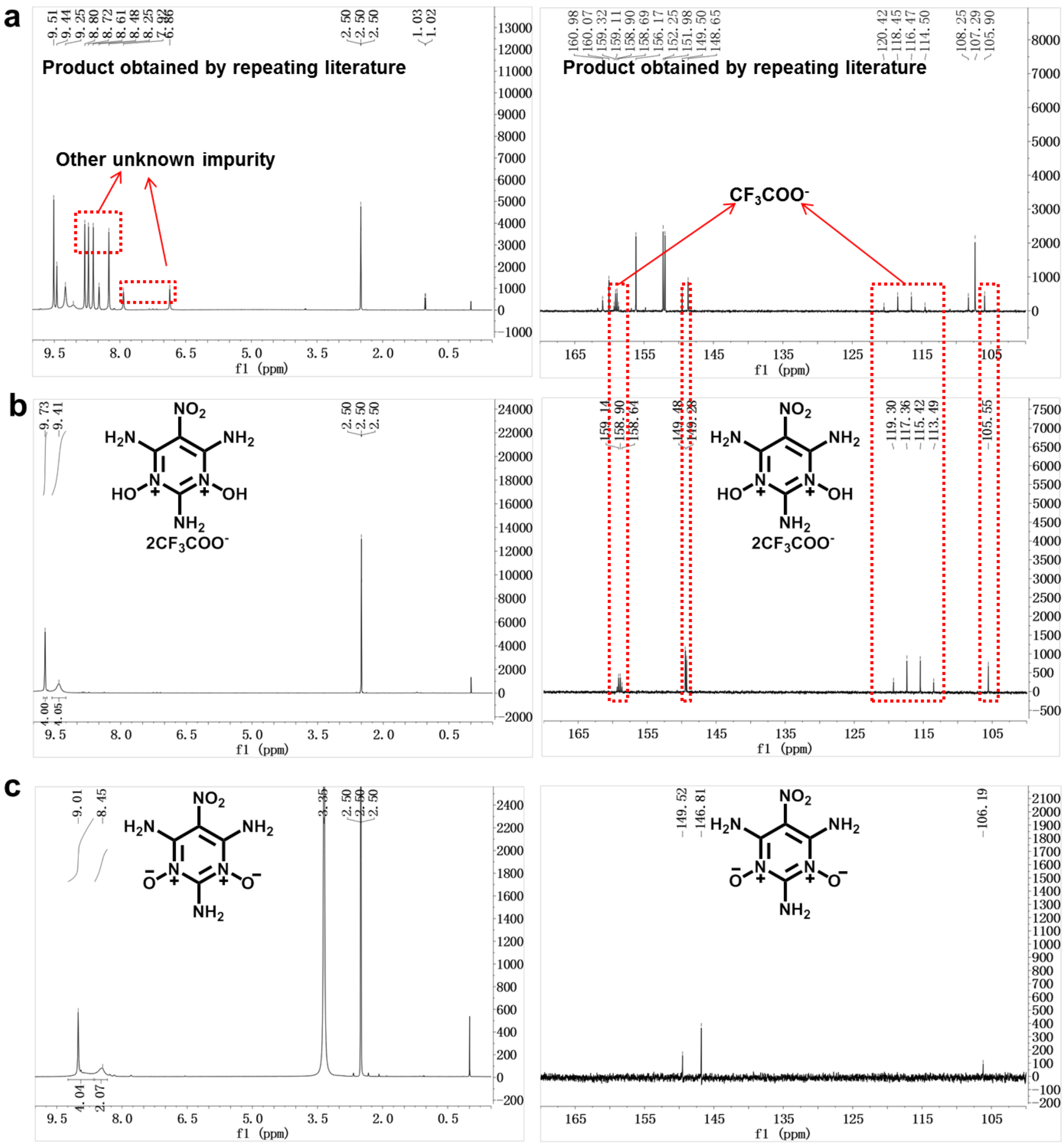

Supplementary Figure 23. a ^{1}H NMR and ^{13}C NMR of the product obtained by repeating literature. **b** ^{1}H NMR and ^{13}C NMR of the single crystal of trifluoroacetate salt of ICM-102. **c** ^{1}H NMR and ^{13}C NMR of pure ICM-102.

Supplementary References

1. Cox, J. D., Wagman, D. D. & Medvedev, V. A. CODATA Key Values for Thermodynamics, Hemisphere Publishing Corp, New York, **1989**.
2. Kissinger, H. E. Reaction Kinetics in Differential Thermal Analysis. *Anal. Chem.* **29**, 1702-1706 (1957).
3. Ozawa, T. A New Method of Analyzing Thermogravimetric Data. *Bull. Chem. Soc. Jpn.* **38**, 1881-1886 (1965).
4. Yi, Q. *et al.* Synthesis, Crystal Structure, and Thermal Behavior of 3-(4-Aminofurazan-3-yl)-4-(4-nitrofurazan-3-yl)furazan (ANTF). *Propellants Explos. Pyrotech.* **41**, 906-911 (2016).
5. Hu, R. Z. *et al.* Thermal Analysis Kinetics, 2nd ed., Science Press, Beijing, 2008 (in Chinese).
6. Gao, Z. *et al.* Synthesis, structural characterization, and thermal properties of a new energetic zinc-FOX-7 complex. *J. Coord. Chem.* **66**, 3572-3580 (2013).
7. Frisch, M. J. *et al.* Gaussian 09, Revision D. 01, Gaussian Inc., Wallingford C T, **2009**.
8. Trouton, F. IV. On molecular latent heat. *Philos. Mag.* **18**, 54-57 (1884).
9. Westwell, M. S., Searle, M. S., Wales, D. J. & Wiliams, D. H. Empirical Correlations between Thermodynamic Properties and Intermolecular Forces. *J. Am. Chem. Soc.* **117**, 5013-5015 (1995).
10. Lu, T., Chen F. Multiwfn: A multifunctional wavefunction analyzer. *J. Comput. Chem.* **33**, 580-592 (2012).
11. Politzer, P. *et al.* An electrostatic interaction correction for improved crystal density prediction. *Mol. Phys.* **107**, 2095-2101 (2009).
12. Rice, B. M. & Byrd, E. F. C. Evaluation of electrostatic descriptors for predicting crystalline density. *J. Comput. Chem.* **34**, 2146-2151 (2013).
13. Lommerse, J. P. M. *et al.* A test of crystal structure prediction of small organic molecules. *Acta Cryst.* **B56**, 697-714 (2000).
14. Mayo, S. L., Olafson, B. D. & Goddard, W. A. III "DREIDING: A generic forcefield", *J. Phys. Chem.* **94**, 8897-8909 (1990).
15. Delia, T. J., Portlock, D. E. & Venton, D. L. Pyrimidine *N*-Oxides. Oxidation of 5-Nitroso-2,4,6-triaminopyrimidine (1). *J. Heterocycl. Chem.* **5**, 449-451 (1968).
16. Hollins, R. A., Merwen, L. H., Nissan, R. A., Wilson, W. S. & Gilardi, R. D. Aminonitroheterocyclic *N*-Oxides--A New Class of Insensitive Energetic Materials. *Mater. Res. Soc. Symp. Proc*. **418**, 31 (1996).
17. Millar, R. W., Philbin, S. P., Claridge, R. P. & Hamid, J. Studies of Novel Heterocyclic Insensitive High Explosive Compounds: Pyridines, Pyrimidines, Pyrazines and Their Bicyclic Analogues. *Propellants Explos. Pyrotech.* **29**, 81-92 (2004).
